# Genomic and Pathogenicity Diversity of Six Avian Reovirus Strains with Different Genotypes

**DOI:** 10.3390/microorganisms14040942

**Published:** 2026-04-21

**Authors:** Xuemei Lu, Guowei He, Jinyang Huang, Ping Liu, Yijian Wu

**Affiliations:** 1 College of Animal Science, Fujian Agriculture and Forestry University, Fuzhou 350002, China; luxuemei2001@163.com (X.L.); heguowei2000@foxmail.com (G.H.); huangjinyang2001@163.com (J.H.); 2 University Key Laboratory for Integrated Chinese Traditional and Western Veterinary Medicine and Animal Healthcare in Fujian Province, Fuzhou 350002, China; 3 Fujian Key Laboratory of Traditional Chinese Veterinary Medicine and Animal Health, Fujian Agriculture and Forestry University, Fuzhou 350002, China

**Keywords:** avian reovirus, genome, sequence analysis, analysis of pathogenicity differences

## Abstract

Avian reovirus (ARV) causes viral arthritis and leads to considerable economic losses in the poultry industry. In this study, six ARV strains of distinct genotypes (FJNP01–FJNP06) were isolated from commercial broiler farms. Through gene sequencing and pathogenicity assessment, we analyzed the genetic evolution and pathogenic characteristics of the σC, P10, σB, μB, and λC genes. Pathogenicity tests revealed that inoculation with FJNP01–FJNP06 by footpad or oral gavage induced symptoms in specific-pathogen-free (SPF) chickens, including mortality and growth retardation. Among the isolates, FJNP04 (genotype IV) showed the highest pathogenicity, causing increased mortality, weight loss, and severe lesions in the footpads and bursa of Fabricius, followed by FJNP05 and FJNP02. The pathogenicity of FJNP06 varied by inoculation route, with enhanced pathogenicity observed following oral gavage. In contrast, FJNP01 and FJNP03 demonstrated relatively low pathogenicity. Identity analysis indicated that σC and P10 were highly variable, σB was relatively conserved, while μB and λC displayed considerable divergence. Phylogenetic analysis placed FJNP01–FJNP06 into genotypes I to VI, respectively, forming six distinct branches on the σC and P10 phylogenetic trees, yet clustering more closely on the σB, μB, and λC trees. The pathogenicity of different genotypes of ARV varies, among which FJNP04 (genotype IV) exhibits the strongest pathogenicity. Genetic sequence analysis revealed that σC and P10 are highly variable, σB is relatively conserved, while μB and λC display a wide range of variation. This study provides insights into the genetic variation and pathogenic characteristics of ARV and serves as a reference for future research.

## 1. Introduction

Avian orthoreovirus (ARV), a member of the family Reoviridae and genus Orthoreovirus, is one of the most significant pathogens affecting the global poultry industry [1,2]. It causes viral arthritis, tenosynovitis, runting–stunting syndrome, respiratory disease, and immunosuppression [2,3,4], resulting in severe economic losses. Viral arthritis and tenosynovitis primarily manifest as swelling of the joints and tendon sheaths in poultry [2], leading to lameness, reduced mobility, and adverse effects on feeding and weight gain. Runting–stunting syndrome is characterized by poor development in young birds and uneven flock growth, with low flock uniformity contributing to economic losses [3]. Respiratory disease presents as coughing, sneezing, and dyspnea, often exacerbated by secondary bacterial or viral infections [4]. Immunosuppression affects the immune organs of poultry, reducing immunity and vaccine efficacy, and increasing susceptibility to secondary infections with other bacteria or viruses [5]. The clinical manifestations of ARV vary, and different genotypes are associated with specific disease symptoms. Specifically, genotype I primarily causes tenosynovitis, arthritis, and malabsorption syndrome, leading to reduced feed conversion efficiency and growth retardation due to lameness and intestinal absorption disorders [4,6,7]. Genotype II mainly induces tenosynovitis, arthritis, and runting–stunting syndrome, affecting flock growth uniformity and resulting in economic losses [4,6]. Genotype III primarily causes tenosynovitis and arthritis, with severe lameness impairing feeding and mobility, thereby inhibiting growth [6,7,8]. Genotype IV exhibits a broad spectrum of clinical manifestations, including tenosynovitis, arthritis, runting–stunting syndrome, and malabsorption syndrome, severely impacting growth and production [4,8,9]. Genotype V mainly leads to tenosynovitis and arthritis, with severe lameness directly limiting feed intake and activity, resulting in impaired weight gain [4,7]. Genotype VI only causes tenosynovitis and arthritis, characterized by joint swelling, heat, and pain, leading to pronounced lameness [8,10]. ARV infects a wide range of hosts and spreads rapidly across broad areas. The transmission routes of ARV mainly include horizontal transmission and vertical transmission, with the former being more common [4]. Horizontal transmission is primarily achieved through contact with feces, respiratory secretions, or contaminated materials (such as drinking water, feed, and litter) from infected birds [11,12]. Furthermore, studies have found that skin wounds on the feet of chicks may serve as a portal of entry for ARV, allowing the virus to breach the body’s barriers and invade the host [4,11]. The virus is highly resistant in the environment and can survive for several weeks to months in poultry farms, forming a persistent source of contamination. Although vertical transmission occurs at a lower rate compared to horizontal transmission, infected breeder chickens can transmit the virus to chicks via eggs, leading to early infection [12]. Although natural mortality rates are relatively low, the lameness, malnutrition, growth retardation, and immunosuppression caused by viral arthritis increase the likelihood of secondary or co-infections, thereby elevating mortality and leading to substantial economic losses in the poultry industry [3,4].

The ARV genome serves as the core carrier of genetic information and function, providing the molecular foundation for studies on viral diversity and genetic evolution. The ARV genome consists of 10 gene segments, which can be broadly classified into three large segments (*L1*, *L2*, *L3*), three medium segments (*M1*, *M2*, *M3*), and four small segments (*S1*, *S2*, *S3*, *S4*) [12,13]. The *L* genes (*L1*, *L2*, *L3*) encode the λ proteins (λA, λB, λC); the *M* genes (*M1*, *M2*, *M3*) encode the μ proteins (μA, μB, μNS); and the *S* genes (*S1*, *S2*, *S3*, *S4*) encode the σ proteins (σC, σA, σB, σNS). Additionally, the *S1* gene also encodes the P10 and P17 proteins [8,12]. The λC protein, encoded by the *L3* gene, is involved in viral attachment to host cells during infection and is critical for the efficiency of viral RNA translation, RNA stability, and immune evasion [8,14]. λC possesses guanylyltransferase activity, and its N-terminal 42 kDa fragment, consisting of approximately 380 amino acid residues, can independently execute self-guanylylation as part of its guanylyltransferase function [14]. The μB protein, encoded by the *M2* gene, is a key component of the viral outer capsid. Its N-terminal myristoylation facilitates interaction with the cell membrane, enabling faster infection of host cells and playing a crucial role in viral entry [15,16]. Cleavage at amino acids 42–43 of the N-terminus generates μBN and μBC, a maturation step during viral assembly, while cleavage at positions 582–583 by trypsin is essential for viral entry [17]. The σB protein, encoded by the *S3* gene, promotes viral binding to receptors, accelerates the infection process, and induces group-specific antibodies against the virus, playing a key role in viral pathogenesis [8,18]. The *S1* gene encodes the p10 protein, a multimeric membrane fusion protein with membrane fusion activity that enhances membrane permeability, induces syncytium formation, and triggers apoptosis, thereby promoting virulence and playing a critical role in viral replication and pathogenicity [8,19]. The ectodomain formed by the N-terminal 1–40 amino acid residues of p10 is a major determinant of differences in fusion efficiency among homologous p10 proteins, within which an N-terminal motif of approximately 25 residues governs the formation of the cysteine loop fusion peptide [20]. The σC protein, also encoded by the *S1* gene, serves as the primary viral cell attachment protein and is a key antigen for inducing specific neutralizing antibodies in the host, playing a central role in viral pathogenicity and immune evasion [19,21]. The major receptor-binding domain of σC is located at residues 151–326 [22], with residues 160–191 forming a triple β-spiral structure and residues 196–326 constituting a β-barrel domain [23]. Among all these proteins, σC exhibits the highest variability and serves as the basis for ARV classification. Based on the σC gene sequence, ARV isolates are typically classified into six genotypes: I, II, III, IV, V, and VI [1,24]. In recent years, with the expansion of intensive farming, the epidemiology of ARV has shown a trend toward increased strain diversity and more complex clinical manifestations, shifting from a predominance of genotype I in previous years to a substantial increase in the proportion of genotypes IV and V in recent years [4,9]. However, the currently widely used classic vaccine strains mainly belong to genotype I, and their nucleotide and amino acid identity with the emerging epidemic genotypes IV and V in the major antigenic protein σC is only around 50–55%, indicating a considerable genetic distance [25]. This significant genetic disparity may lead to the inability of vaccine-induced neutralizing antibodies to effectively recognize and neutralize variant strains. Consequently, vaccine failure or reduced protective efficacy may occur.

In this study, we conducted pathogenicity tests in SPF chickens using six different genotypes of ARV strains isolated and identified from a large-scale white feather broiler farm. Chickens were infected via two routes: footpad and oral gavage. Footpad was used to simulate direct tissue infection and induction of tenosynovitis, while oral gavage was used to mimic the natural horizontal transmission route via the digestive tract. The results showed that all six ARV strains caused growth inhibition and other symptoms in the chicks, and different infection routes exhibited distinct pathogenicity. Furthermore, we sequenced and analyzed the σC, P10, σB, μB, and λC proteins of ARV, which are closely involved in virus–host interactions. The results showed that σC and P10 genes were highly variable, the σB gene was relatively conserved, while the μB and λC genes displayed a wide range of variation.

## 2. Materials and Methods

### 2.1. Materials

Six different genotypes of ARV were isolated, identified, and preserved by our laboratory from joint tendon and synovial fluid samples collected from clinically affected chickens suspected of ARV infection in a large-scale white feather broiler farm. The samples were homogenized, filtered, and inoculated into SPF chicken embryos for virus isolation and amplification. After five blind passages, the isolates were confirmed to be free of exogenous pathogen contamination through routine purity testing. Viral RNA was extracted, and the σC gene was amplified by RT-PCR and sequenced. Genotyping of the isolates was performed based on phylogenetic analysis of the σC gene sequences according to established classification criteria. Finally, six ARV strains were obtained and designated as FJNP01, FJNP02, FJNP03, FJNP04, FJNP05, and FJNP06, with genotypes I, II, III, IV, V, and VI, respectively. After genotyping, the passaged virus samples were collected and stored at −80 °C. SPF chicken embryonated eggs were purchased from Xinjiang Dahuanong Poultry Egg Co., Ltd. (Xinxing, China). SPF chickens were hatched in our laboratory. The Animal Virus DNA/RNA Rapid Extraction Kit was purchased from Tianlong Technology (Xi’an) Co., Ltd. (Xi’an, China). The HiScript II One Step RT-PCR Kit and HiScript II One Step qRT-PCR SYBR Green Kit were purchased from Vazyme Biotech (Nanjing) Co., Ltd. (Nanjing, China).

### 2.2. Virus Amplification

The virus strains were filtered through a 0.22 μm microporous membrane for sterilization purposes and inoculated into 7-day-old SPF chicken embryos via the yolk sac route. The inoculated embryos were incubated at 37 °C and observed every 12 h. Embryos dying within 24 h were promptly discarded. Allantoic fluid, embryo bodies, and chorioallantoic membranes from embryos that died after 24 h were collected, placed into a high-throughput tissue grinder, and ground at 30 oscillations per minute for 3 min per cycle, repeated twice. The collected samples were aliquoted.

### 2.3. ELD_50_ Determination

Forty-two 7-day-old SPF chicken embryos were randomly assigned to 7 groups (6 embryos per group). The fifth-passage virus samples were filtered through a 0.22 μm microporous membrane for sterilization. Serial 10-fold dilutions (10^−3^, 10^−4^, 10^−5^, 10^−6^, 10^−7^, and 10^−8^) of the virus samples were prepared using sterile PBS and inoculated into the SPF chicken embryos in each group via the yolk sac route. The seventh group served as the blank control, with 6 SPF chicken embryos inoculated with an equal volume of sterile PBS. All groups were incubated at 37 °C and observed every 24 h, with embryo mortality recorded at each time point. Mortality was recorded from 24 to 168 h post-inoculation. The 50% embryo lethal dose (ELD_50_) of the virus was calculated using the Reed–Muench method.

### 2.4. Pathogenicity Test

A total of 112 1-day-old healthy SPF chickens were randomly divided into 7 groups of 16 chicks each. Experimental groups 1–6 were designated as Groups A, B, C, D, E, and F, respectively. Group A SPF chickens were infected with strain FJNP01; Group B with FJNP02; Group C with FJNP03; Group D with FJNP04; Group E with FJNP05; and Group F with FJNP06. Based on previous studies [9,26] and the experimental conditions of this study, eight chicks in each group were inoculated via the footpad route, and the other eight via the oral gavage route. The inoculation dose for each chick in groups 1–6 was 0.2 mL (containing 10^3^ ELD_50_). Group 7 served as the control group, with 16 1-day-old healthy SPF chickens inoculated with an equal volume of sterile PBS via both footpad and oral gavage routes. After challenge, all chicks were housed in isolators. The health status of chicks in the experimental groups was observed daily, and morbidity was recorded based on the occurrence of footpad and joint swelling. Chick mortality was also recorded. Body weight of each SPF chick in all groups was measured every 3 days, and individual body weight data were recorded. Cloacal swabs were collected from SPF chickens in each group every 4 days to detect viral shedding and analyze shedding patterns. Surviving SPF chickens in each group were weighed on day 27, then euthanized and subjected to necropsy. The spleen and bursa were collected, weighed, and the bursa-to-body weight ratio (BFW) and spleen-to-body weight ratio (SBR) were calculated. Tissues including the heart, liver, spleen, duodenum, bursa, cecal tonsils, footpads, and hock joints were collected for viral load detection and quantification. Statistical analyses for survival rate, body weight change, bursa/body weight ratio, spleen/body weight ratio, cloacal swab viral shedding, and tissue viral load were performed using GraphPad Prism 10.1.2 software. Quantitative data were expressed as mean ± SD. Survival curves were analyzed using the Kaplan–Meier method with the log-rank test. Body weight change was analyzed using repeated measures ANOVA. Comparisons of bursa/body weight ratio, spleen/body weight ratio, cloacal swab viral shedding, and tissue viral load were performed using one-way ANOVA with Tukey’s test.

### 2.5. Gene Amplification and Sequencing

RNA was extracted from the fifth-passage virus samples following the instructions of the Animal Virus DNA/RNA Rapid Extraction Kit. Using the extracted RNA as a template, ARV was amplified with the HiScript II One Step RT-PCR Kit according to the primers listed in the reference (Table 1) [8]. The RT-PCR products were recovered and sent to Shanghai Bioengineering Co., Ltd. (Shanghai, China) for sequencing analysis.

### 2.6. Sequence Analysis

The sequencing results were assembled using the Seqman Pro 11.1.0 module of DNAStar. The assembled sequences were then aligned with ARV reference strains (Table 2) from NCBI for nucleotide and amino acid sequence identity analysis using MegAlign 11.1.0 software. Amino acid sequence alignment was performed using ESPript 3.2. Maximum likelihood phylogenetic trees were constructed using MEGA11.0 software, with branch support calculated through 1000 bootstrap replicates to generate the phylogenetic trees.

## 3. Results

### 3.1. ELD_50_ Detection

The ELD_50_ of ARV isolates FJNP01, FJNP02, FJNP03, FJNP04, FJNP05, and FJNP06 was determined using 42 7-day-old SPF chicken embryos per strain and calculated by the Reed–Muench method. The results showed that the ELD_50_ of FJNP01 was 10^−4.80^/200 μL, FJNP02 was 10^−3.70^/200 μL, FJNP03 was 10^−5.50^/200 μL, FJNP04 was 10^−5.64^/200 μL, FJNP05 was 10^−3.87^/200 μL, and FJNP06 was 10^−3.40^/200 μL.

### 3.2. Pathogenicity Analysis

#### 3.2.1. Survival Rate of SPF Chickens

**Figure 1:**
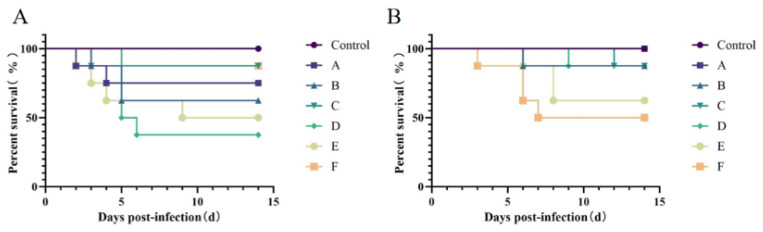
Survival rates of SPF chickens infected with FJNP01–FJNP06 isolates (n = 8 chickens per group). (**A**) SPF chickens infected by footpad. (**B**) SPF chickens infected via oral gavage. Survival curves were analyzed using the Kaplan–Meier method.

For chicks infected via the footpad route (Figure 1A), mortality peaked during the first 5 days, and no further mortality was observed after day 10. For chicks infected via oral gavage (Figure 1B), mortality peaked between days 6 and 9 and stabilized after day 14. Except for Group A (oral gavage), which showed no mortality, all challenged groups exhibited differences in mortality compared with the control group. In the footpad groups, mortality rates ranked from highest to lowest were D, E, B, A, F, C. In the oral gavage groups, mortality rates ranked from highest to lowest were F, E, D, B, C, A. Notably, mortality rates in footpad-inoculated Groups D and E were ≥50%, while mortality in oral gavage Group F was also ≥50%. These results indicate that FJNP04 and FJNP05 are highly lethal to SPF chickens by footpad, whereas FJNP06 exhibits high lethality via oral gavage.

#### 3.2.2. Body Weight Changes of SPF Chickens

**Figure 2:**
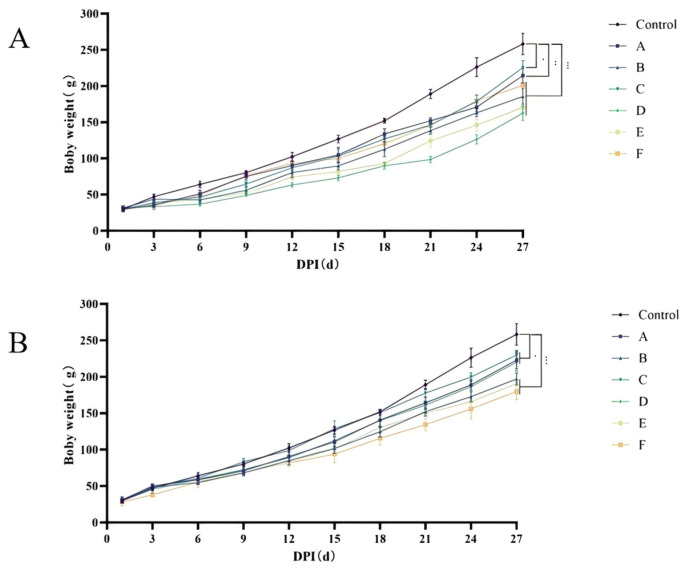
Body weight change curves of SPF chickens infected with FJNP01–FJNP06 isolates (n = 8 chickens per group). (**A**) SPF chickens infected by footpad. (**B**) SPF chickens infected via oral gavage. * *p* < 0.05, ** *p* < 0.01, *** *p* < 0.001 indicate statistically significant differences compared with the control group.

The results of body weight changes in SPF chickens are shown in Figure 2. At each time point, the body weight of the blank control group was consistently higher than that of all challenged groups (A–F), with the weight gap progressively widening over time. Among footpad-inoculated groups (Figure 2A), Group D exhibited the slowest weight gain, followed by Groups E, B, F, A, and C. Among oral gavage-inoculated groups (Figure 2B), Group F exhibited the slowest weight gain, followed by Groups E, B, D, A, and C. These results indicate that infection with FJNP01–FJNP06 significantly suppressed body weight gain in SPF chickens. Specifically, FJNP04, FJNP05, FJNP02, and FJNP06 caused severe weight suppression following footpad, while FJNP06, FJNP05, and FJNP02 caused severe weight suppression following oral gavage, leading to a significant decline in the growth performance of infected chickens.

#### 3.2.3. Morbidity Rate of SPF Chickens

The morbidity results of SPF chickens are shown in Table 3, indicated that the morbidity rate of SPF chickens inoculated via the footpad route was significantly higher than that of chickens inoculated via oral gavage. In the footpad-inoculated groups, Groups D and E began showing symptoms at 3 days post-infection (dpi), all groups exhibited symptoms by 9 dpi, and 100% morbidity was achieved in all groups by 15 dpi. In contrast, in the oral gavage-inoculated groups, Group F showed symptoms at 3 dpi, all groups exhibited symptoms by 21 dpi, and only Group E reached 100% morbidity by 27 dpi.

#### 3.2.4. Gross Pathological Observation of SPF Chickens

**Figure 3:**
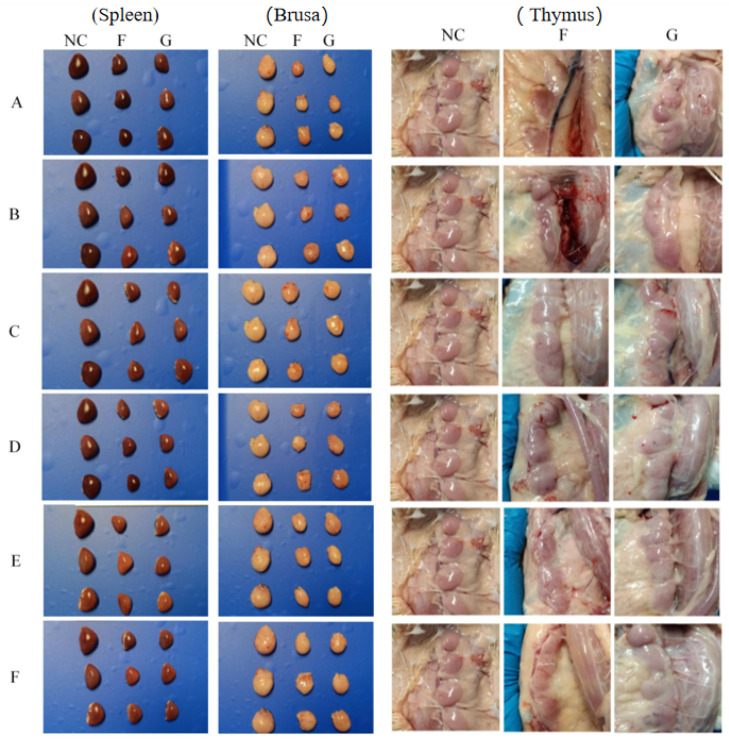
Pathological changes in the spleen, bursa, and thymus of SPF chickens infected with FJNP01–FJNP06 isolates at 27 days post-infection. Superscript NC indicates blank control, F indicates footpad, and G indicates oral gavage. (**A**) FJNP01. (**B**) FJNP02. (**C**) FJNP03. (**D**) FJNP04. (**E**) FJNP05. (**F**) FJNP06.

**Figure 4:**
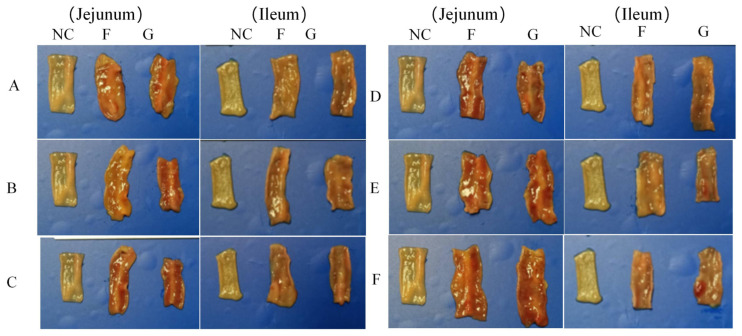
Jejunal and ileal lesions in SPF chickens infected with FJNP01–FJNP06 isolates at 27 days post-infection. Superscript NC indicates blank control, F indicates footpad, and G indicates oral gavage. (**A**) FJNP01. (**B**) FJNP02. (**C**) FJNP03. (**D**) FJNP04. (**E**) FJNP05. (**F**) FJNP06.

All chickens in the blank control group remained healthy, showing no clinical symptoms or pathological changes (Figure 3 and Figure 4 NC control group). In contrast, SPF chickens inoculated with FJNP01–FJNP06 via either footpad or oral gavage showed marked atrophy of the spleen and bursa, as well as mild diffuse or petechial hemorrhage in the thymus (Figure 3). Regarding intestinal pathology, footpad inoculation with FJNP01–FJNP06 induced diffuse hemorrhage in the jejunum and ileum of SPF chickens (Figure 4), with Groups D, E, and F exhibiting more severe ileal hemorrhage. Following oral gavage, jejunal and ileal hemorrhage with thinning of the intestinal wall was also observed (Figure 4). Groups A, B, C, D, and E showed diffuse hemorrhage, while Group F exhibited dense petechial hemorrhage in the jejunum and massive hemorrhage in the ileum.

#### 3.2.5. Immune Organ Index Determination in SPF Chickens

**Figure 5:**
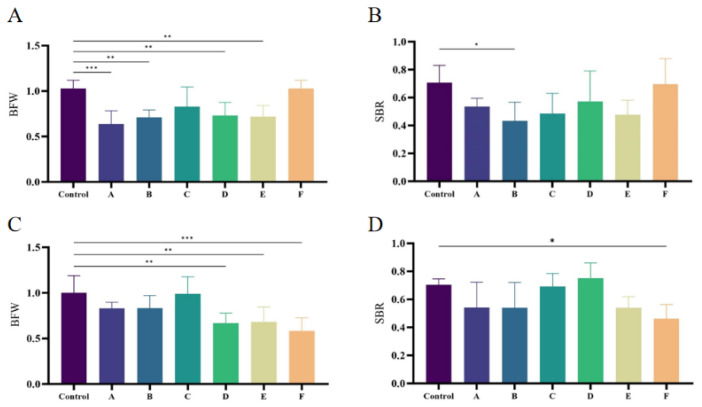
Bursa-to-body weight ratio (BFW) and spleen-to-body weight ratio (SBR) of SPF chickens infected with FJNP01–FJNP06 isolates. (**A**) BFW of chickens infected by footpad. (**B**) SBR of chickens infected by footpad. (**C**) BFW of chickens infected via oral gavage. (**D**) SBR of chickens infected via oral gavage. Data are presented as mean ± SD (n = 8 chickens per group). The control group values for BFW and SBR are presented as the raw ratio to body weight. * *p* < 0.05, ** *p* < 0.01, *** *p* < 0.001 indicate statistically significant differences compared with the control group.

The results of immune organ indices of SPF chickens are shown in Figure 5, compared with the blank control group, chickens in all experimental groups exhibited varying degrees of difference in BFW. Specifically, chickens in footpad-inoculated Group A showed highly significant differences in BFW compared with the control group (*p* < 0.001), followed by Groups B, E, and D, which also showed significant differences (*p* < 0.01). Among oral gavage-inoculated groups, chickens in Group F showed highly significant differences in BFW compared with the control group (*p* < 0.001), followed by Groups E and D, which also showed significant differences (*p* < 0.01). In contrast, differences in SBR between the experimental groups and the control group were less pronounced for both inoculation routes. Only footpad-inoculated Group B and oral gavage-inoculated Group F showed significant differences in SBR compared with the control group (*p* < 0.05).

#### 3.2.6. Viral Shedding in SPF Chickens

**Figure 6:**
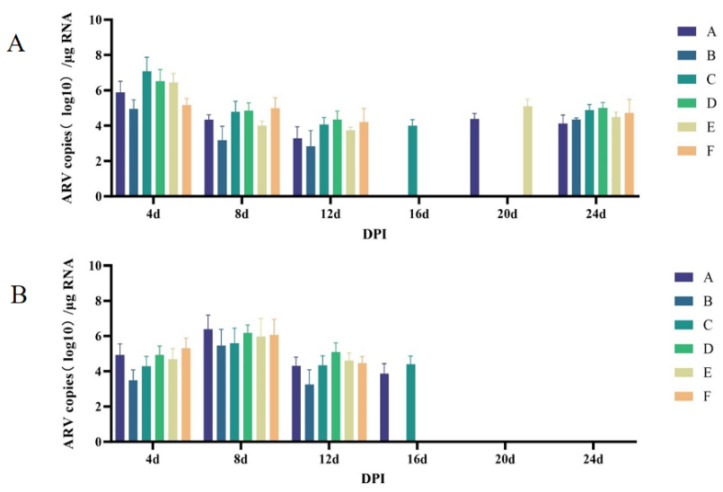
Viral shedding detection in cloacal swabs of SPF chickens infected with FJNP01–FJNP06 isolates. (**A**) SPF chickens infected by footpad. (**B**) SPF chickens infected via oral gavage. Data are presented as mean ± SD (n = 8 chickens per group).

The results of viral shedding detection in cloacal swabs of SPF chickens are shown in Figure 6A. Viral shedding was detected in swabs from all footpad-inoculated groups by 4 days post-infection (dpi), with shedding levels peaking at this time point and with high initial levels that subsequently declined. Shedding ceased in all groups except Group C by 16 dpi. A second round of shedding was detected in swabs from Groups A and E at 20 dpi, and by 24 dpi, viral shedding was detected in all groups, with shedding levels comparable to those observed at 8 dpi. Figure 6B shows that viral shedding was also detected in swabs from all oral gavage-inoculated groups by 4 dpi. However, in contrast to footpad-inoculated groups, shedding levels peaked at 8 dpi in oral gavage-inoculated groups, exhibiting a pattern of low–high–low. No second round of shedding was observed in SPF chickens inoculated via oral gavage.

#### 3.2.7. Viral Load Detection in Various Tissues and Organs of SPF Chickens

**Figure 7:**
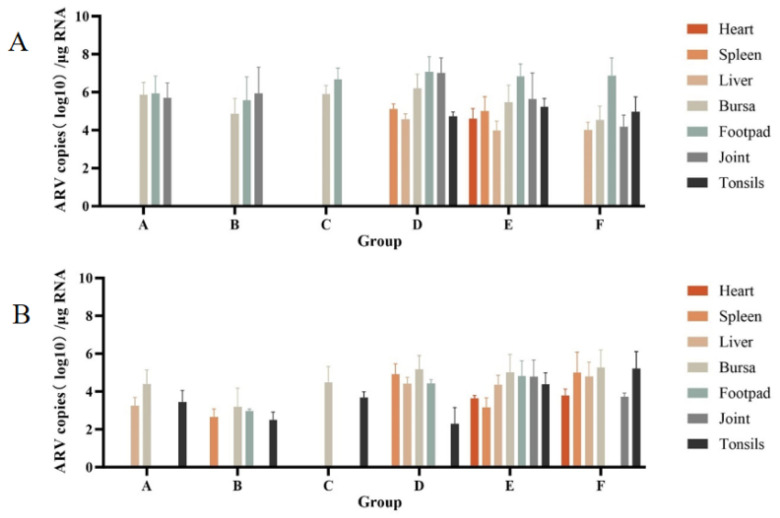
Viral load detection in various tissues and organs of SPF chickens infected with FJNP01–FJNP06 isolates. (**A**) SPF chickens infected by footpad. (**B**) SPF chickens infected via oral gavage. Data are presented as mean ± SD (n = 8 chickens per group).

The results of viral load detection in various tissues and organs of SPF chickens are shown in Figure 7, regardless of inoculation route (footpad or oral gavage), FJNP01–FJNP06 exhibited broad tissue tropism. Notably, fluorescent signals were detected in both footpad and bursa samples from all footpad-inoculated groups. Viral loads in footpads ranked from highest to lowest were Groups D, F, E, C, A, and B. Viral loads in the bursa ranked from highest to lowest were Groups D, C, A, E, B, and F. In oral gavage-inoculated groups, fluorescent signals were detected in both cecal tonsils and bursa samples from all groups. Viral loads in cecal tonsils ranked from highest to lowest were Groups F, E, C, A, B, and D. Viral loads in the bursa ranked from highest to lowest were Groups F, D, E, C, A, and B. These findings indicate that target organs may vary depending on the inoculation route. However, regardless of the route, the bursa was consistently identified as a key target organ for FJNP01–FJNP06.

#### 3.2.8. Summary of Pathogenicity Analysis

FJNP01–FJNP06 induced tenosynovitis, arthritis, and growth inhibition in chickens via both footpad and oral gavage routes. With the exception of FJNP01, all strains caused mortality in chicks. Notably, FJNP04 and FJNP05 achieved ≥50% mortality following footpad, while FJNP06 achieved ≥50% mortality following oral gavage. Regarding effects on immune organs, all strains except FJNP03 caused varying degrees of decline in immune organ indices. Viral tissue load analysis revealed that the bursa was a key target organ regardless of inoculation route. Comprehensive pathogenicity assessment indicated that FJNP04 exhibited the strongest pathogenicity, followed by FJNP05 and FJNP02. Of note, FJNP06 showed weak pathogenicity by footpad but showed greater pathogenicity following oral gavage, whereas FJNP01 and FJNP03 exhibited relatively weak pathogenicity via both inoculation routes.

### 3.3. Sequence Identity Analysis

In the σC identity analysis (Supplementary Figure S1), FJNP01 showed the highest identity with A-chicken-526-China-2014 (nt: 71.5%, aa: 76.6%). FJNP02 showed high identity with both A-chicken-PHC-China-2022 and A-chicken-202305-China-2024 (nt: 96.8–97.0%, aa: 97.1–97.7%). FJNP03 showed the highest identity with A-chicken-01224A-USA-2016 (nt: 63.2%, aa: 67.9%). FJNP04 showed the highest identity with A-chicken-D4-Canada-2020 (nt: 88.2%, aa: 88.6%). FJNP05 showed the highest identity with both A-chicken-94594-USA-2023 and A-chicken-S5-USA-2024 (nt: 99.7%, aa: 99.0%). FJNP06 showed the highest identity with A-chicken-3211-Hungary-2016 (nt: 85.2%, aa: 86.0%). Additionally, identity between FJNP01–FJNP06 and the classical vaccine strain S1133 ranged from nt 43.5–70.7% and aa 45.1–73.4%. Identity among FJNP01–FJNP06 strains ranged from nt 43.1–59.9% and aa 46.1–63.0%. The substantial divergence among FJNP01–FJNP06 strains and their low identity with the classical vaccine strain S1133 suggest that classical vaccines may not provide complete protection against FJNP01–FJNP06.

We also analyzed the identity of P10, σB, μB, and λC among FJNP01–FJNP06. P10 showed considerable divergence (nt: 59.3–76.0%, aa: 60.6–83.0%). σB was relatively conserved (nt: 88.2–97.3%, aa: 94.6–99.5%). μB exhibited broad identity ranges (nt: 71.8–97.9%, aa: 88.0–97.9%), with FJNP02 and FJNP03 showing relatively lower identity with FJNP01, FJNP04, FJNP05, and FJNP06 (71.8% ≤ nt ≤ 73.1%, 88.0% ≤ aa ≤ 89.1%). λC also exhibited broad identity ranges (nt: 72.2–99.1%, aa: 83.4–99.2%), primarily due to FJNP03 being distinct from the other strains (72.2% ≤ nt ≤ 72.6%, 83.4% ≤ aa ≤ 83.9%). Apart from σC and P10, identity analysis of σB, μB, and λC generally showed the highest pairwise identity among FJNP01–FJNP06 strains.

### 3.4. Phylogenetic Tree Analysis

**Figure 8:**
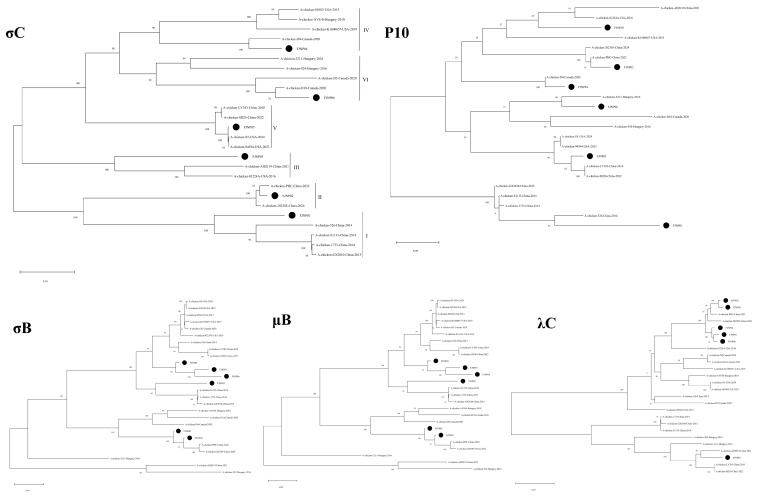
Phylogenetic trees of FJNP01–FJNP06 isolates constructed using the maximum likelihood method in MEGA11.0 based on σC, P10, σB, μB, and λC proteins. FJNP01–FJNP06 isolates are indicated by black circles.

To further analyze the genetic evolution of FJNP01–FJNP06, phylogenetic analysis of the σC, P10, σB, μB, and λC nucleotide sequences was performed using MEGA11.0 (Figure 8). In the σC phylogenetic tree, FJNP01 clustered within genotype I along with S1133, 1733, 526, and GX2010, showing close genetic relatedness to A-chicken-526-China-2014. FJNP02 clustered within genotype II along with PHC and 202305, sharing a sub-branch with A-chicken-PHC-China-2022, indicating close genetic relatedness. FJNP03 clustered within genotype III along with AHZJ19 and 01224A. FJNP04 clustered within genotype IV along with K1600657, 05682, D4, and AVS-B, sharing a sub-branch with A-chicken-D4-Canada-2020, indicating close genetic relatedness. FJNP05 clustered within genotype V along with LY383, S5, 94594, and SD26, showing closer genetic relatedness to A-chicken-94594-USA-2023 and A-chicken-S5-USA-2024. FJNP06 clustered within genotype VI along with 3211, D10, D2, and 924, sharing a sub-branch with A-chicken-3211-Hungary-2016. The genetic relationships shown by the P10 phylogenetic tree were generally consistent with those of σC. In the σB phylogenetic tree, FJNP01–FJNP06 were relatively clustered. In the μB phylogenetic tree, FJNP01, FJNP04, FJNP05, and FJNP06 were relatively clustered, while FJNP02 and FJNP03 clustered together on a separate branch. In the λC phylogenetic tree, FJNP01, FJNP02, FJNP04, FJNP05, and FJNP06 clustered together on one branch, while FJNP03 stood independently. These results are consistent with the identity analysis findings.

### 3.5. Unique Amino Acids

Identity and phylogenetic analyses have clarified the classification and genetic relationships of FJNP01–FJNP06. To characterize the amino acid variations among FJNP01–FJNP06, we performed amino acid sequence alignments of σC, P10, σB, μB, and λC. These alignments revealed several amino acid mutations in FJNP01–FJNP06 that distinguished them from the reference strains. Specifically, FJNP01–FJNP06 were found to possess 34, 11, 37, 22, 12, and 16 unique amino acids, respectively, among which 11, 8, 11, 5, 9, and 7 sites, respectively, represented relatively conserved positions exhibiting hydrophilic/hydrophobic property reversals. Detailed sites are shown in Table 4. Notably, FJNP01 and FJNP03 shared 23 and 24 unique amino acids in σC, respectively, with identical mutations at positions 7, 42, 87, 94, 101, 113, 122, and 256 that distinguished them from the reference strains. Among these, position 256 is located within the major receptor-binding domain.

Additionally, several amino acid mutation hotspots were identified. Due to the extremely high variability of σC, its relatively conserved regions were also documented (Table 5). σC exhibited 10 consecutive hypervariable sites and 8 consecutive conserved sites. λC exhibited 3 consecutive hypervariable sites, while P10, σB, and μB showed no consecutive hypervariable sites.

## 4. Discussion

In recent years, the endemic regions of ARV have continued to expand, and the emergence of variant strains has become increasingly frequent, causing substantial economic losses to the poultry industry. Currently, vaccination remains the primary strategy for ARV prevention and control. However, the increasing number of viral arthritis cases caused by ARV variants in poultry farms across multiple regions of China in recent years [25] suggests that existing commercial vaccines may only provide partial protection against continuously emerging novel ARV strains [24,25]. Although several ARV strains have been identified and characterized, knowledge regarding the genetic diversity and pathogenic characteristics of ARV strains circulating in China remains limited. To address this gap, the present study conducted pathogenicity tests in SPF chickens using six ARV strains of different genotypes isolated from the Fujian region of China between 2023 and 2024. Additionally, sequencing and comparative analyses were performed on the σC, P10, σB, μB, and λC proteins. These proteins are closely involved in host interactions.

ARV infection exhibits considerable diversity in clinical manifestations, and the association between different genotypes and specific disease syndromes has become a focus of research. Regardless of genotype, tenosynovitis and arthritis are the most common clinical presentations, which may also result in lameness, runting–stunting syndrome, growth inhibition, and immunosuppression [4]. Previous studies have shown that the pathogenicity of different genotypes of ARV varies significantly, with genotypes I and IV having higher global prevalence, but their clinical manifestations are not consistent [4,9]. Genotype IV exhibits the most diverse clinical manifestations, including tenosynovitis/arthritis, runting–stunting syndrome, malabsorption syndrome, and other symptoms, posing diagnostic challenges. In contrast, genotype VI shows a relatively focused clinical presentation, mainly tenosynovitis/arthritis [1,6]. However, systematic comparative studies on the pathogenic characteristics of different ARV genotypes circulating in the field in China remain limited. Moreover, most existing studies have used a single infection route (footpad), and it remains unclear whether the pathogenic performance of different genotypes is consistent under natural infection routes such as oral infection [25]. Therefore, this study systematically compared the pathogenicity of six different genotypes of ARV isolates via two routes: footpad and oral gavage.

In this study, six different genotypes of avian reovirus (FJNP01–FJNP06) inoculated by footpad and oral gavage both induced significant growth inhibition in SPF chickens, and the body weights of all challenged groups were significantly lower than those of the blank control group. All chickens inoculated via the footpad exhibited footpad or joint swelling. In contrast, for chickens inoculated via oral gavage, only those infected with FJNP04, FJNP05, and FJNP06 showed a high morbidity rate (≥75%), whereas the morbidity rates caused by the other isolates were ≤37.5%. All isolates caused mortality in infected chicks, among which FJNP04 and FJNP05 by footpad led to ≥50% mortality, and FJNP06 via oral gavage resulted in ≥50% mortality. All challenged groups showed depression, growth retardation, as well as atrophy of the spleen and bursa, and thymus hemorrhage. In the footpad groups, ileal hemorrhage was more severe in chicks infected with FJNP04, FJNP05, and FJNP06; the oral gavage groups generally showed diffuse intestinal hemorrhage, among which the FJNP06 group displayed dense punctate hemorrhage in the jejunum and massive hemorrhage in the ileum. Except for FJNP03, the other isolates reduced the immune organ indices of chickens to varying degrees, indicating a certain immunosuppressive effect. Viral shedding patterns showed that after footpad, all isolates reached peak shedding at 4 days post-infection, and shedding basically ceased by 16 days except for FJNP03, but a second round of shedding occurred at 20–24 days. After oral gavage, the peak of viral shedding appeared at 8 days, and shedding stopped completely after 16 days with no second round observed. Tissue viral load detection revealed that after footpad, all isolates could be detected in the footpad and bursa, among which FJNP04 showed the highest viral load in both tissues. After oral gavage, all isolates were detected in the bursa and cecal tonsils, among which FJNP06 exhibited the highest viral load in both tissues. The results indicated that the bursa is an important target organ of ARV regardless of the infection route.

Based on comprehensive evaluation of multiple indicators including chick survival rate, growth inhibition, gross lesions, viral shedding patterns, and tissue viral load, FJNP04 (genotype IV) showed the strongest pathogenicity. The finding that genotype IV is highly pathogenic is consistent with the results of several previous studies [4,9], followed by FJNP05 (genotype V) and FJNP02 (genotype II). Notably, FJNP06 (genotype VI) showed weak pathogenicity by footpad, but its pathogenic characteristics were significantly enhanced after oral gavage, with strong pathogenicity in terms of mortality and growth inhibition. In contrast, FJNP01 (genotype I) and FJNP03 (genotype III) showed relatively weak overall pathogenicity via both inoculation routes. However, these differences are route-dependent phenomena under experimental challenge models and only reflect the pathogenic performance of different genotypes under specific artificial infection routes. The pathogenicity ranking of the six ARV isolates was determined by comprehensive assessment of multiple indices, each independently assessed and then integrated to form the overall relative virulence profile. Since a single indicator cannot fully reflect viral pathogenicity, the overall virulence hierarchy was determined by comprehensively analyzing all measured outcomes. This study is exploratory in nature, aiming to compare the pathogenic characteristics of different ARV genotypes circulating in the field in China.

The σC protein mediates viral attachment to host cells and induces the production of neutralizing antibodies. Therefore, it is a critical protein for ARV infection, host immune responses, and vaccine development [19,24]. Driven by high variability under vaccine immune pressure, σC serves as the basis for ARV genotyping [25], with previous studies classifying ARV into six genotypes based on σC, significant antigenic differences among different genotypes lead to reduced or even complete loss of protective efficacy of traditional vaccine strains against some circulating strains [1,24]. In this study, σC analysis classified FJNP01, FJNP02, FJNP03, FJNP04, FJNP05, and FJNP06 as genotypes I, II, III, IV, V, and VI, respectively, with nucleotide and amino acid identity among isolates ranging from 43.1% to 58.3% and 46.1% to 62.7%, respectively. Identity with the vaccine strain S1133 was also low (nt 43.5–70.7%; aa 45.1–73.4%). This highlights the high variability of the σC protein. This extreme variability underlies the utility of σC for genotyping, and is likely associated with antigenic drift, which may limit the protective efficacy of traditional vaccines.

The P10 protein possesses membrane fusion activity and plays a key role in viral replication and pathogenicity [19]. Sequence analysis revealed that P10 also exhibits high variability, with genetic relationships generally consistent with those of σC. This concordance suggests that P10 and σC may evolve under similar selective pressures, but functional linkage remains to be determined. The σB protein induces group-specific antibodies against the virus and plays a key role in viral pathogenesis [18,27]. Identity analysis, phylogenetic trees, and sequence alignments indicated that σB is relatively conserved, suggesting that σB may be involved in maintaining core pathogenic functions. Both μB and λC play crucial roles in viral entry into host cells [8,14], but identity analysis revealed broad divergence, consistent with previous findings by Kumar [28] showing higher variability in *M2* and *L3* beyond the *S1* gene. Notably, FJNP03 clustered with FJNP02 in the μB phylogenetic tree but was distantly related to the other five strains in the λC tree. Such differences may lead to varying outcomes during host entry or adaptation to different environments. Furthermore, while the genetic evolution of σC and P10 was relatively consistent, the evolution of σB, μB, and λC appeared to occur independently via different pathways, which may indicate possible genetic reassortment events, although formal recombination analysis is needed for confirmation. Genetic reassortment is a key mechanism in ARV evolution, potentially generating novel ARV genotypes with distinct genetic profiles in chickens [9]. Moreover, reassortment could modify the combination of the virus’s major antigenic protein σC with other internal genes, enabling circulating strains to alter their antigenicity while retaining their original pathogenicity, thus completely breaching the immunological barrier established by traditional vaccines such as S1133, and evading the existing immunity conferred by vaccination.

Amino acid sequence alignments of σC, P10, σB, μB, and λC revealed that FJNP01–FJNP06 possessed 34, 11, 37, 22, 12, and 16 unique amino acid mutations, respectively, among which 11, 8, 11, 5, 9, and 7 sites exhibited amino acid property reversals. This variability may contribute to strain-specific adaptations, but its impact on pathogenicity requires functional validation.

This study also has certain limitations. First, the pathogenicity tests were conducted only in SPF chickens with a relatively small sample size (n = 8), which to some extent limits the generalizability of the conclusions. Second, only one isolate per genotype was included in this study, and thus the results cannot be directly generalized to all strains of the same genotype. Significant genetic diversity and inter-strain differences may exist within each genotype; therefore, the comparisons of pathogenicity among genotypes presented in this study are all based on individual strains and may not represent all circulating strains of the same genotype. Moreover, this study only observed some characteristic amino acid differences without further elucidating their molecular functions. Future research should expand the host range and sample size, include more isolates per genotype, and further investigate the key amino acid residues determining tissue tropism and virulence, as well as the mechanisms of virus–host immune system interactions.

In summary, this study preliminarily explored the pathogenicity phenotypes of six different ARV genotypes, obtained the σC, P10, σB, μB, and λC gene sequences of each strain, and systematically analyzed their homology and genetic evolutionary relationships. Information regarding ARV pathogenicity and genetic diversity is critical for developing more effective vaccines and control strategies, and this study provides a reference for improving disease prevention, surveillance, and management.

## Figures and Tables

**Table 1:** Primer information for amplification of avian reovirus genes.

Primer Name	Sequence (5′ → 3′)	Product Size/bp
S1-F	GCTTTTTCAGTCCTTCGTGTCAATGTT	1643
S1-R	GATGAATAACCAGTCCCCTTA	
S3-F	GCTTTTTGAGTCCTTAGCGT	1202
S3-R	GATGAATAGGCGAGTCCCGCTA	
M2-F	GCTTTTTCAGTGCCAATCTTTCTCA	2158
M2-R	GATGAATAACGTGCCAATCC	
L3-F	GCTTTTTCACCCATGGCTCAGATTA	3907
L3-R	GATGAGTAACACCCTTCTACT	

**Table 2:** The 20 ARV reference strains used in the analysis were obtained from the GenBank database.

No.	Strain	Country	Year	σC-Based Genotype	GenBank Number			
					σC, P10	σB	μB	λC
1	S1133	China	2014	I	KF741762.1	KF741764.1	KF741760.1	KF741758.1
2	1733	China	2014	I	KF741712.1	KF741714.1	KF741710.1	KF741708.1
3	526	China	2014	I	KF741702.1	KF741704.1	KF741700.1	KF741698.1
4	GX/2010/1	China	2015	I	KJ476705.1	KJ476707.1	KJ476703.1	KJ476701.1
5	PHC-2020-0545	China	2022	II	MW174790.1	MW174792.1	MW174788.1	MW174786.1
6	HN/WLK/202305	China	2024	II	PQ490783.1	PQ490785.1	PQ490781.1	PQ490779.1
7	AHZJ19	China	2021	III	OK077999.1	OK078003.1	OK077997.1	OK077995.1
8	Reo/PA/Layer/01224A/14	USA	2016	III	KT428304.1	KT428306.1	KT428302.1	KT428300.1
9	K1600657	USA	2019	IV	MK583337.1	MK583339.1	MK583335.1	MK583333.1
10	Reo/PA/Broiler/05682/12	USA	2015	IV	KM877331.1	KM877333.1	KM877329.1	KM877327.1
11	D4	Canada	2020	IV	MN879630.1	MN879632.1	MN879628.1	MN879626.1
12	AVS-B	Hungary	2010	IV	FR694197.1	FR694199.1	FR694195.1	FR694193.1
13	LY383	China	2018	V	MF183217.1	MF183219.1	MF183215.1	MF183213.1
14	TRV-S5	USA	2024	V	PP657725.1	PP657771.1	PP657730.1	PP657728.1
15	ARV_94594	USA	2023	V	OR612139.1	OR612141.1	OR612137.1	OR612135.1
16	V-ARV-SD26	China	2022	V	MW244848.1	MW244850.1	MW244846.1	MW244844.1
17	3211-V-02	Hungary	2016	VI	KX398278.1	KX398280.1	KX398276.1	KX398274.1
18	D10	Canada	2020	VI	MN879690.1	MN879692.1	MN879688.1	MN879686.1
19	D2	Canada	2020	VI	MN879610.1	MN879612.1	MN879608.1	MN879606.1
20	924-Bi-05	Hungary	2016	VI	KX398268.1	KX398270.1	KX398266.1	KX398264.1
21	FJNP01	China	2023	I	PZ112175	PZ112177	PZ112173	PZ112171
22	FJNP02	China	2024	II	PZ112185	PZ112187	PZ112183	PZ112181
23	FJNP03	China	2024	III	PZ112195	PZ112197	PZ112193	PZ112191
24	FJNP04	China	2023	IV	PZ112205	PZ112207	PZ112203	PZ112201
25	FJNP05	China	2023	V	PZ112215	PZ112217	PZ112213	PZ112211
26	FJNP06	China	2024	VI	PZ112225	PZ112227	PZ112223	PZ112221

**Table 3:** Morbidity rate of SPF chickens infected with FJNP01–FJNP06 isolates, defined by the occurrence of swelling in the footpads or joints.

Group	Morbidity Rate (%)				
	3 Dpi	9 Dpi	15 Dpi	21 Dpi	27 Dpi
Control	0.0	0.0	0.0	0.0	0.0
Footpad-A	0.0	100.0	100.0	100.0	100.0
Oral gavage-A	0.0	0.0	12.5	12.5	25.0
Footpad-B	0.0	87.5	100.0	100.0	100.0
Oral gavage-B	0.0	12.5	12.5	37.5	37.5
Footpad-C	0.0	75.0	100.0	100.0	100.0
Oral gavage-C	0.0	0.0	0.0	12.5	12.5
Footpad-D	12.5	100.0	100.0	100.0	100.0
Oral gavage-D	0.0	12.5	37.5	50.0	75.0
Footpad-E	25.0	100.0	100.0	100.0	100.0
Oral gavage-E	0.0	37.5	50.0	87.5	100.0
Footpad-F	0.0	75.0	100.0	100.0	100.0
Oral gavage-F	12.5	62.5	75.0	75.0	87.5

**Table 4:** Unique amino acids in the amino acid sequences of FJNP01–FJNP06 isolates compared with reference strains. Asterisks (*) indicate mutations at relatively conserved sites that result in hydrophilic/hydrophobic property reversals.

Strain	Unique Amino Acid Sites				
	σC	P10	σB	μB	λC
**FJNP01**	7(L *) 42(V) 87(A) 94(T) 101(D) 102(E) 112(G) 113(M *) 122(N) 123(E) 147(N) 155(D *) 199(K) 203(V) 255(N) 256(M) 285(A) 310(N) 313(S *)	31(N)	166(S) 354(C *) 355(R) 357(A *)	19(N) 23(C) 29(P *) 38(H *) 638(A *) 665(S *) 666(Y *) 667(R)	812(R) 1246(C)
**FJNP02**		27(W *) 53(Q *)	290(A *) 353(R) 354(C *) 355(R) 357(A *)	653(V *) 665(S *)	438(F *) 1075(R)
**FJNP03**	7(L *) 42(K) 47(M) 76(V) 80(S) 87(A) 94(E) 101(N) 105(L) 110(Q) 111(E) 113(R) 122(K) 133(H) 150(D) 151(T) 159(A) 163(M) 169(T) 171(Q *) 186(T) 252(F) 256(Q) 300(S *)	31(N) 53(T *) 59(S) 86(A *)	353(R) 354(C *) 355(R) 357(A *)	437(S *) 653(V *)	200(F) 273(P *) 1164(A *)
**FJNP04**	77(L) 105(V) 118(V) 146(D) 153(K) 186(Y)	32(H *) 37(T) 48(A) 52(V) 65(M *) 71(H) 77(L)	354(A) 355(E) 357(A)	460(K) 461(H)	204(Y) 422(T *) 754(T *) 1075(R)
**FJNP05**			155(H) 353(A *) 354(C *) 355(R) 357(A *)	73(M *) 268(A *) 543(A *) 660(A *) 665(S *) 666(N *) 667(E) 668(E)	438(F *)
**FJNP06**	77(E) 106(D) 120(N) 144(N) 171(N *) 234(V)	35(S) 52(V) 83(S *)	290(A *) 307(T *) 353(R) 354(C *) 355(R) 357(A *)		626(M *)

**Table 5:** Special amino acid sites in the σC, P10, σB, μB, and λC proteins of ARV. Criteria for identification: consecutive hypervariable or conserved regions are defined as three or more consecutive amino acid sites exhibiting hypervariability or conservation.

Protein	Consecutive Hypervariable Regions	Conserved Regions
**σC**	69–71, 75–79, 83–85, 94–96, 118–120, 122–124, 156–159, 184–186, 254–256, 309–313	8–13, 15–21, 173–175, 178–183, 188–191, 227–229, 238–240, 274–276
**P10**		
**σB**		
**μB**		
**λC**	48–50, 316–318, 464–466	

## Data Availability

The genome sequences of FJNP01–FJNP06 obtained in this study have been deposited in GenBank, and their accession numbers are listed in Table 2.

## Supplementary Materials

The following supporting information can be downloaded at: https://www.mdpi.com/article/10.3390/microorganisms14040942/s1, Figure S1: Nucleotide and amino acid sequence identity analysis of FJNP01–FJNP06 isolates based on σC, P10, σB, μB, and λC proteins compared with reference strains.
