# Supplementary material for: Genomic and Pathogenicity Diversity of Six Avian Reovirus Strains with Different Genotypes
Source: Microorganisms. 2026 Apr 21;14(4):942. doi: 10.3390/microorganisms14040942 (PMC13118900; doi:10.3390/microorganisms14040942)
Supplement: Supplementary file 1 [file microorganisms-14-00942-s001.zip › microorganisms-4221493-supplementary.pdf]

Supplementary Figure S1. Nucleotide and amino acid sequence identity analysis of FJNP01–FJNP06 isolates based on σC, P10, σB, μB, and λC proteins compared with reference strains.

(1-a) σC Nucleotide sequence identity analysis

Percent Identity (upper triangle); Divergence (lower triangle)

	1	2	3	4	5	6	7	8	9	10	11	12	13	14	15	16	17	18	19	20	21	22	23	24	25	26		
1		98.9	79.1	98.5	50.7	50.2	49.7	48.3	43.7	43.8	43.9	43.9	44.8	45.3	45.3	44.8	45.2	43.8	40.9	44.9	70.7	50.4	46.1	43.9	45.2	43.5	1	A-chicken-S1133-China-2014
2	1.0		79.1	98.6	50.3	49.8	49.3	47.8	43.4	43.7	43.8	43.9	44.5	45.2	45.2	44.5	44.9	43.9	41.0	44.9	70.3	50.1	45.7	43.6	45.1	43.2	2	A-chicken-1733-China-2014
3	21.6	21.8		78.5	50.1	49.9	49.6	48.3	42.8	42.8	45.1	43.0	46.8	46.5	46.5	46.8	46.2	43.5	39.9	45.2	71.5	49.3	44.9	44.4	46.6	44.6	3	A-chicken-526-China-2014
4	1.3	1.2	22.1		49.6	49.1	48.9	47.9	43.6	43.7	43.8	43.5	44.4	45.0	45.0	44.4	44.8	43.8	40.9	45.3	70.4	49.4	45.8	43.1	44.9	43.2	4	A-chicken-GX2010-China-2015
5	59.2	59.5	60.2	59.7		96.8	47.5	45.8	46.2	46.3	48.2	45.9	47.6	47.8	47.8	47.6	46.6	48.9	46.7	46.8	48.5	96.8	45.0	46.8	47.7	45.3	5	A-chicken-PHC-China-2022
6	59.6	59.9	59.6	60.1	3.2		47.6	46.3	46.6	46.3	48.4	45.8	47.2	47.5	47.5	47.2	45.9	48.8	46.2	46.5	48.6	97.0	46.2	46.9	47.4	45.1	6	A-chicken-202305-China-2024
7	64.3	64.8	64.4	65.7	64.8	65.1		80.8	46.8	47.2	51.0	48.2	48.3	47.8	47.8	48.3	51.0	50.1	47.0	51.5	48.0	46.7	61.0	49.8	47.9	48.4	7	A-chicken-AHZJ19-China-2021
8	68.6	69.2	65.9	69.5	68.6	68.0	20.1		47.7	47.4	52.9	48.0	47.9	48.2	48.2	47.9	48.4	49.9	47.8	48.3	45.6	46.6	63.2	53.0	48.1	48.5	8	A-chicken-01224A-USA-2016
9	71.2	71.5	74.6	70.8	72.3	71.7	67.1	67.9		83.0	61.0	83.6	57.2	56.4	56.4	57.3	59.0	53.2	56.0	59.1	44.4	46.2	48.4	61.4	56.5	56.4	9	A-chicken-K1600657-USA-2019
10	70.1	70.4	73.9	70.1	73.1	74.2	66.6	67.2	17.6		61.6	92.8	57.3	57.2	57.2	57.3	61.2	55.1	60.6	57.0	45.7	45.7	49.5	62.8	57.3	57.9	10	A-chicken-05682-USA-2015
11	70.6	70.0	72.3	70.9	69.7	70.7	62.2	59.4	45.1	42.4		61.4	57.6	57.3	57.3	57.7	60.8	57.4	54.8	57.1	44.9	47.6	51.2	88.2	57.4	59.5	11	A-chicken-D4-Canada-2020
12	71.0	71.3	73.4	71.8	73.0	74.8	65.1	66.4	17.5	7.0	43.5		58.4	58.6	58.6	58.3	60.9	55.8	59.5	57.0	43.9	45.6	50.2	63.0	58.7	57.2	12	A-chicken-AVS-B-Hungary-2010
13	70.6	70.9	68.7	71.2	70.8	72.2	66.1	66.4	50.7	51.1	49.2	48.0		96.2	96.2	99.8	55.6	55.5	51.1	58.5	45.2	46.9	49.4	57.3	96.2	53.7	13	A-chicken-LY383-China-2018
14	70.4	70.7	69.3	71.0	70.9	72.3	65.3	65.0	51.2	50.2	49.5	47.4	3.7		99.9	96.2	54.9	54.2	50.3	58.2	44.2	46.9	49.9	57.4	99.7	52.9	14	A-chicken-S5-USA-2024
15	70.4	70.7	69.3	71.0	70.9	72.3	65.3	65.0	51.2	50.2	49.5	47.4	3.7	0.0		96.2	54.9	54.2	50.3	58.2	44.2	46.9	49.9	57.4	99.7	52.9	15	A-chicken-94594-USA-2023
16	70.6	70.9	68.6	71.2	70.8	72.1	66.0	66.4	50.5	51.1	49.0	48.2	0.1	3.7	3.7		55.5	55.5	51.1	58.5	45.2	46.9	49.3	57.4	96.2	53.7	16	A-chicken-SD26-China-2022
17	67.9	68.8	69.1	68.8	69.9	72.8	60.2	64.1	48.2	44.1	44.8	45.0	50.0	52.5	52.5	50.2		64.5	57.9	69.3	45.6	46.5	48.2	61.8	54.8	85.2	17	A-chicken-3211-Hungary-2016
18	71.3	71.3	77.3	71.3	68.6	69.6	63.1	63.3	57.8	54.4	50.7	53.4	53.5	56.5	56.5	53.4	40.5		81.9	62.7	45.4	48.3	51.0	57.6	54.3	62.5	18	A-chicken-D10-Canada-2020
19	79.6	79.3	86.6	79.3	72.4	73.4	65.9	63.6	51.1	42.2	52.5	45.3	59.8	61.5	61.5	59.8	48.6	16.0		57.3	41.6	45.1	46.7	54.5	50.4	55.1	19	A-chicken-D2-Canada-2020
20	71.1	72.1	74.1	70.5	65.5	67.0	59.0	64.1	48.1	50.5	50.3	50.0	46.2	47.4	47.4	46.3	33.9	41.1	47.9		44.1	46.4	49.6	57.8	58.1	68.1	20	A-chicken-924-Hungary-2016
21	31.5	31.5	31.7	31.4	58.3	57.2	65.9	68.5	68.4	68.4	70.1	68.9	66.5	67.1	67.1	66.5	64.7	67.7	78.1	70.1		48.8	45.2	43.1	44.1	45.8	21	FJNP01
22	60.5	60.8	59.3	60.9	3.2	3.0	66.0	68.0	71.0	74.8	71.9	74.4	71.5	71.6	71.6	71.4	72.4	69.5	74.4	67.0	58.5		44.4	46.4	46.8	44.4	22	FJNP02
23	68.9	69.8	69.7	69.8	68.1	67.2	43.5	40.9	65.4	63.0	59.6	62.6	60.7	61.0	61.0	60.9	65.4	61.2	65.4	61.1	68.8	71.0		52.5	49.6	48.6	23	FJNP03
24	71.8	72.1	72.8	73.3	72.7	73.7	65.2	60.8	45.0	42.0	12.1	42.2	50.4	50.5	50.5	50.2	44.3	51.8	54.5	49.4	70.9	74.0	59.6		57.7	59.9	24	FJNP04
25	71.0	71.3	69.3	71.6	71.5	72.9	65.3	65.0	51.2	50.2	49.5	47.4	3.7	0.2	0.2	3.7	52.5	56.5	61.5	47.4	67.7	72.2	61.5	50.0		52.8	25	FJNP05
26	71.3	71.9	72.0	71.7	72.4	74.4	64.1	63.7	50.7	48.7	46.2	49.6	52.8	54.6	54.6	52.8	14.6	40.8	50.2	33.8	66.0	75.0	62.2	47.0	54.6		26	FJNP06
	1	2	3	4	5	6	7	8	9	10	11	12	13	14	15	16	17	18	19	20	21	22	23	24	25	26		

(1-b) σC amino sequence identity analysis

Percent Identity (upper triangle); Divergence (lower triangle)

	1	2	3	4	5	6	7	8	9	10	11	12	13	14	15	16	17	18	19	20	21	22	23	24	25	26		
1		97.4	80.5	96.8	55.5	54.9	51.0	49.4	46.8	46.4	47.7	46.1	46.8	47.1	47.1	46.8	47.7	48.4	45.1	44.2	73.4	55.2	48.7	47.4	46.8	45.1	1	A-chicken-S1133-China-2014
2	2.3		79.9	97.1	55.2	54.5	50.6	48.7	46.4	46.1	48.1	45.8	46.4	46.4	46.4	46.4	47.7	48.4	45.1	43.8	72.7	54.9	48.4	47.1	46.1	45.1	2	A-chicken-1733-China-2014
3	22.3	23.1		79.9	54.9	54.2	51.3	48.4	47.1	47.1	46.8	47.1	47.4	48.4	48.4	47.4	48.1	48.4	45.1	45.8	76.6	54.5	47.1	46.4	48.7	45.8	3	A-chicken-526-China-2014
4	3.0	2.7	23.1		53.9	53.2	50.0	48.7	46.4	45.8	47.7	45.5	46.4	46.8	46.8	46.4	47.4	48.4	45.1	44.5	72.7	53.9	48.4	46.8	46.4	45.5	4	A-chicken-GX2010-China-2015
5	65.4	66.2	66.9	69.2		96.4	49.0	48.1	49.4	49.7	51.3	50.3	50.3	50.3	50.3	50.3	47.4	49.4	46.1	48.4	52.9	97.7	47.1	49.4	50.0	48.1	5	A-chicken-PHC-China-2022
6	66.9	67.7	68.4	70.7	3.3		49.7	48.4	49.4	50.0	51.3	50.6	50.0	49.7	49.7	50.0	47.1	49.4	45.8	48.7	53.2	97.1	47.7	49.7	49.4	48.1	6	A-chicken-202305-China-2024
7	74.7	75.5	73.9	77.2	81.2	79.4		84.1	49.7	50.6	55.5	51.6	50.6	49.7	49.7	50.6	53.2	51.9	50.6	50.3	51.3	49.0	67.5	55.2	49.7	51.0	7	A-chicken-AHZJ19-China-2021
8	78.5	80.3	81.2	80.3	84.4	82.6	17.2		50.6	51.0	55.5	51.3	50.0	49.0	49.0	50.0	52.9	51.0	50.0	48.7	49.4	48.7	67.9	55.5	49.0	51.0	8	A-chicken-01224A-USA-2016
9	88.5	89.5	87.6	89.5	80.7	80.7	79.0	76.0		87.7	67.9	87.7	59.1	58.8	58.8	59.1	64.9	60.4	62.0	61.4	47.7	49.7	51.6	66.2	58.4	62.3	9	A-chicken-K1600657-USA-2019
10	91.4	92.4	87.6	92.4	79.9	79.0	76.4	75.1	13.2		67.5	91.6	58.4	57.5	57.5	58.4	65.6	58.8	62.3	61.0	48.7	50.0	51.3	65.6	57.1	64.0	10	A-chicken-05682-USA-2015
11	85.2	84.3	88.1	85.2	75.2	75.2	64.1	64.1	41.1	41.7		68.5	60.4	59.4	59.4	60.4	64.9	61.4	59.4	63.6	47.1	51.0	54.9	88.6	59.4	63.0	11	A-chicken-D4-Canada-2020
12	90.4	91.4	87.6	92.4	78.1	77.3	73.9	74.3	13.2	8.6	40.0		58.4	58.1	58.1	58.4	66.6	60.4	62.3	63.3	47.7	50.6	52.9	67.9	57.8	65.3	12	A-chicken-AVS-B-Hungary-2010
13	88.1	89.0	86.2	89.0	77.7	78.5	76.8	78.1	57.6	59.0	54.6	59.0		95.5	95.5	99.7	59.4	58.4	54.9	59.4	48.1	49.0	51.6	61.0	95.1	57.1	13	A-chicken-LY383-China-2018
14	87.1	89.0	83.4	88.1	77.7	79.4	79.4	80.7	58.3	61.1	56.6	59.7	4.4		99.7	95.5	58.1	56.5	53.6	58.1	48.1	48.7	51.3	60.4	99.0	55.5	14	A-chicken-S5-USA-2024
15	87.1	89.0	83.4	88.1	77.7	79.4	79.4	80.7	58.3	61.1	56.6	59.7	4.4	0.0		95.5	58.1	56.5	53.6	58.1	48.1	48.7	51.3	60.4	99.0	55.5	15	A-chicken-94594-USA-2023
16	88.1	89.0	86.2	89.0	77.7	78.5	76.8	78.1	57.6	59.0	54.6	59.0	0.0	4.4	4.4		59.4	58.4	54.9	59.4	48.1	49.0	51.6	61.0	95.1	57.1	16	A-chicken-SD26-China-2022
17	85.2	85.2	84.3	86.2	85.7	86.7	71.0	71.8	46.3	45.1	46.3	43.4	56.6	59.3	59.3	56.6		68.5	61.7	75.0	47.7	47.4	52.9	65.6	57.8	86.0	17	A-chicken-3211-Hungary-2016
18	83.0	83.0	83.0	83.0	79.9	79.9	73.9	76.0	55.2	57.9	52.2	54.6	59.0	63.2	63.2	59.0	39.3		82.1	69.5	48.4	49.4	52.9	61.4	56.2	66.6	18	A-chicken-D10-Canada-2020
19	89.3	89.3	89.3	89.3	85.9	87.8	73.4	74.6	48.6	48.0	53.3	48.0	64.0	67.0	67.0	64.0	48.8	17.6		61.7	45.1	45.8	49.7	58.8	53.2	59.1	19	A-chicken-D2-Canada-2020
20	92.8	93.8	87.8	91.8	81.7	81.7	78.1	81.6	50.2	50.9	45.9	46.5	55.9	58.6	58.6	55.9	27.6	37.0	47.6		45.5	48.1	52.6	63.3	57.8	75.0	20	A-chicken-924-Hungary-2016
21	32.2	33.2	27.3	33.2	72.0	71.2	75.6	80.3	85.2	82.5	86.7	85.2	84.3	84.3	84.3	84.3	84.8	83.4	90.2	92.0		52.9	47.4	46.1	47.7	48.0	21	FJNP01
22	66.2	66.9	67.7	69.2	2.0	2.7	82.1	82.6	79.9	79.0	76.0	77.3	81.2	82.1	82.1	81.2	85.7	79.9	87.8	82.6	72.0		47.1	49.0	48.4	47.4	22	FJNP02
23	80.3	81.2	84.9	81.2	88.2	85.4	41.7	40.7	75.1	75.1	64.4	70.2	73.9	74.7	74.7	73.9	70.6	71.1	76.3	71.5	86.7	87.3		55.8	51.3	51.6	23	FJNP03
24	86.2	87.1	89.0	88.1	80.3	79.4	64.8	64.1	43.9	45.1	11.7	41.1	53.3	54.6	54.6	53.3	45.1	52.2	54.7	46.5	89.5	81.2	62.9		60.7	63.0	24	FJNP04
25	88.1	90.0	82.5	89.0	78.5	80.3	79.4	80.7	59.0	61.8	56.6	60.4	4.7	0.7	0.7	4.7	60.0	63.9	67.8	59.3	85.2	83.0	74.7	53.9		55.2	25	FJNP05
26	93.0	93.0	91.0	92.0	85.7	85.7	75.6	76.0	51.1	48.1	49.5	45.7	61.8	65.4	65.4	61.8	14.5	43.4	55.2	29.2	84.9	87.6	73.5	49.5	66.2		26	FJNP06
	1	2	3	4	5	6	7	8	9	10	11	12	13	14	15	16	17	18	19	20	21	22	23	24	25	26		

(1-c) σB Nucleotide sequence identity analysis

Percent Identity

Divergence

	1	2	3	4	5	6	7	8	9	10	11	12	13	14	15	16	17	18	19	20	21	22	23	24	25	26		
1		99.5	84.2	98.9	72.7	72.5	60.9	83.3	83.6	83.9	72.1	72.7	83.7	83.7	83.8	83.6	68.8	71.9	83.7	59.6	84.0	72.5	72.2	83.9	84.9	83.1	1	A-chicken-S1133-China-2014
2	0.5		84.3	98.9	72.6	72.4	61.1	83.4	83.7	83.9	72.1	72.8	83.7	83.7	83.8	83.7	69.1	72.0	83.8	60.0	84.2	72.4	72.1	84.1	85.0	83.2	2	A-chicken-1733-China-2014
3	16.9	16.8		84.2	73.2	73.1	61.1	92.8	93.5	94.1	73.1	72.8	93.3	93.8	93.9	93.2	70.4	73.0	93.6	58.9	86.8	73.4	73.0	89.6	83.3	86.3	3	A-chicken-526-China-2014
4	1.1	1.1	16.8		72.6	72.4	60.6	83.0	83.4	83.6	72.2	72.8	83.7	83.4	83.5	83.7	68.7	71.9	83.5	59.5	83.9	72.5	72.2	83.7	84.7	82.9	4	A-chicken-GX2010-China-2015
5	30.2	30.4	29.7	30.2		98.8	59.8	74.2	73.9	73.8	80.9	79.6	72.9	73.5	73.5	73.0	71.9	79.6	74.1	61.3	73.3	96.9	95.3	73.9	72.3	73.8	5	A-chicken-PHC-China-2022
6	30.4	30.6	29.8	30.3	1.2		59.9	74.0	73.7	73.6	80.5	79.1	72.7	73.3	73.3	72.7	72.0	79.6	73.9	61.3	73.2	96.2	94.5	73.8	72.4	73.6	6	A-chicken-202305-China-2024
7	44.2	44.1	43.9	44.4	46.8	46.2		60.9	61.0	61.0	60.4	59.4	60.8	60.7	60.9	60.9	62.2	60.4	61.3	80.4	61.0	59.6	60.1	60.9	59.6	60.4	7	A-chicken-AHZJ19-China-2021
8	18.3	18.2	7.3	18.6	28.6	28.8	44.0		96.6	97.1	72.9	73.4	90.5	96.7	96.8	90.6	70.4	73.2	96.8	59.5	84.6	74.0	73.5	89.7	81.6	85.8	8	A-chicken-01224A-USA-2016
9	18.0	17.8	6.5	18.2	29.0	29.2	44.0	3.5		99.1	73.1	72.7	90.8	98.7	98.9	90.8	69.7	73.1	98.7	59.2	85.3	74.0	73.5	90.7	82.5	86.5	9	A-chicken-K1600657-USA-2019
10	17.6	17.6	6.0	18.0	29.1	29.4	43.8	2.9	0.8		73.1	73.0	91.3	99.4	99.5	91.3	70.3	73.6	99.3	59.0	85.4	73.7	73.4	91.0	82.5	86.7	10	A-chicken-05682-USA-2015
11	30.8	30.9	29.6	30.6	21.2	21.6	43.9	30.3	29.9	29.9		88.5	74.0	72.6	72.7	74.1	71.9	86.5	72.9	60.3	73.4	80.6	81.9	73.3	71.4	73.6	11	A-chicken-D4-Canada-2020
12	29.9	30.0	29.6	29.6	22.7	23.1	46.2	29.3	30.1	29.8	12.3		73.4	72.6	72.6	73.5	71.3	88.4	72.8	59.3	72.6	79.7	81.6	72.3	71.8	72.2	12	A-chicken-AVS-B-Hungary-2010
13	17.7	17.7	7.0	17.5	30.3	30.6	44.4	10.0	9.8	9.2	28.5	29.1		90.9	91.0	99.9	71.0	72.7	91.0	58.8	86.1	73.0	72.6	88.5	83.3	86.2	13	A-chicken-LY383-China-2018
14	18.0	17.9	6.3	18.3	29.6	29.8	44.2	3.4	1.3	0.6	30.5	30.2	9.6		99.6	90.9	70.0	73.3	98.8	58.4	85.1	73.4	73.0	90.4	82.0	86.2	14	A-chicken-S5-USA-2024
15	17.8	17.7	6.2	18.1	29.4	29.7	43.8	3.2	1.1	0.5	30.3	30.0	9.5	0.4		91.0	70.2	73.4	99.0	58.7	85.2	73.5	73.0	90.6	82.1	86.3	15	A-chicken-94594-USA-2023
16	17.8	17.7	7.1	17.5	30.2	30.5	44.1	10.0	9.8	9.3	28.3	29.0	0.1	9.7	9.5		70.9	72.6	91.1	58.8	86.1	73.0	72.7	88.4	83.1	86.2	16	A-chicken-SD26-China-2022
17	34.6	34.4	32.2	34.6	30.6	30.7	41.6	32.2	32.9	32.3	31.4	31.9	31.4	32.8	32.5	31.5		73.1	70.5	60.1	69.8	71.2	71.6	70.3	69.4	71.0	17	A-chicken-3211-Hungary-2016
18	31.0	31.1	30.1	31.1	22.6	22.6	45.1	29.6	29.7	29.2	14.4	12.3	30.1	29.7	29.5	30.1	29.3		73.6	61.8	73.3	79.0	80.4	73.2	72.2	72.5	18	A-chicken-D10-Canada-2020
19	17.8	17.7	6.5	18.1	28.8	29.0	43.5	3.3	1.3	0.7	30.2	30.0	9.5	1.2	1.0	9.5	32.2	29.3		59.3	85.0	73.9	73.5	90.4	82.0	86.5	19	A-chicken-D2-Canada-2020
20	45.2	45.0	45.9	45.3	43.9	43.7	21.9	46.1	46.2	46.6	44.6	45.9	46.5	47.4	47.1	46.4	45.2	43.2	45.9		58.6	60.4	60.5	58.9	59.3	59.8	20	A-chicken-924-Hungary-2016
21	16.9	16.7	13.9	17.0	29.2	29.3	44.3	16.2	15.5	15.5	29.4	30.2	14.7	15.9	15.6	14.8	33.1	29.7	15.9	46.5		72.9	72.1	93.3	83.2	93.0	21	FJNP01
22	30.5	30.6	29.3	30.2	3.1	3.8	47.1	29.0	28.9	29.1	21.5	22.2	30.0	29.6	29.4	29.9	31.7	23.3	28.9	45.2	29.7		97.9	73.3	72.4	73.1	22	FJNP02
23	30.8	30.9	29.8	30.6	4.8	5.5	46.4	29.5	29.3	29.4	19.8	19.6	30.4	30.0	29.9	30.3	31.3	21.5	29.3	45.2	30.6	2.0		73.0	71.8	72.6	23	FJNP03
24	17.1	16.9	10.6	17.1	28.4	28.5	43.7	10.4	9.2	8.9	29.3	30.6	12.0	9.5	9.3	12.1	32.1	29.7	9.6	45.6	6.2	29.1	29.4		83.2	91.4	24	FJNP04
25	16.4	16.3	18.3	16.6	30.8	30.6	44.7	20.3	19.4	19.4	31.9	31.2	17.9	20.0	19.8	18.1	33.9	31.1	19.9	45.7	18.4	30.4	31.1	17.9		82.9	25	FJNP05
26	18.4	18.4	14.6	18.5	28.7	29.0	45.3	15.2	14.5	14.4	29.3	30.9	15.2	15.0	14.8	15.1	31.5	30.4	14.6	45.0	7.1	29.6	30.1	8.6	18.9		26	FJNP06
	1	2	3	4	5	6	7	8	9	10	11	12	13	14	15	16	17	18	19	20	21	22	23	24	25	26		

(1-d) σB amino sequence identity analysis

Percent Identity

Divergence

	1	2	3	4	5	6	7	8	9	10	11	12	13	14	15	16	17	18	19	20	21	22	23	24	25	26		
1		99.1	94.1	98.5	88.6	88.2	70.9	94.4	94.2	94.4	88.9	88.6	94.4	93.9	94.1	94.5	85.7	88.2	94.1	71.0	93.2	88.2	88.2	94.5	94.7	94.7	1	A-chicken-S1133-China-2014
2	0.7		94.2	98.7	88.8	88.3	71.0	94.5	94.4	94.5	89.1	88.8	94.5	94.1	94.2	94.7	85.8	88.3	94.2	71.2	93.4	88.3	88.3	94.8	94.8	94.8	2	A-chicken-1733-China-2014
3	6.0	5.9		93.6	88.2	87.7	70.0	96.5	97.0	97.2	88.6	88.8	97.9	96.6	96.8	97.8	86.1	88.6	96.9	70.0	94.4	87.7	87.6	96.2	94.2	95.9	3	A-chicken-526-China-2014
4	1.3	1.2	6.5		88.6	88.2	70.8	93.9	93.8	93.9	88.9	88.5	93.9	93.5	93.6	94.1	85.5	88.2	93.6	70.9	92.8	88.2	88.2	94.1	94.2	94.2	4	A-chicken-GX2010-China-2015
5	12.2	12.0	12.7	12.2		99.4	70.6	88.8	89.1	88.9	94.5	93.9	89.1	88.3	88.5	89.2	87.6	94.1	88.9	71.2	88.8	98.5	98.4	89.5	89.1	89.8	5	A-chicken-PHC-China-2022
6	12.7	12.6	13.3	12.7	0.4		70.5	88.3	88.6	88.5	94.1	93.5	88.6	87.9	88.0	88.8	87.4	93.6	88.5	70.8	88.3	98.1	97.9	89.1	88.6	89.4	6	A-chicken-202305-China-2024
7	35.1	34.8	36.5	35.3	35.8	36.0		70.6	70.3	70.5	70.2	70.6	70.2	70.0	70.2	70.3	72.4	70.2	70.8	91.8	69.9	70.2	70.3	70.0	69.6	70.6	7	A-chicken-AHZJ19-China-2021
8	5.7	5.5	3.5	6.2	12.0	12.6	35.5		98.4	98.5	89.1	89.2	97.0	97.9	98.1	97.2	86.1	88.0	98.5	70.5	94.1	88.3	88.2	96.0	94.4	95.7	8	A-chicken-01224A-USA-2016
9	5.9	5.7	2.9	6.3	11.7	12.2	36.0	1.5		99.4	89.2	89.4	97.6	98.8	99.0	97.8	85.7	88.3	99.1	70.2	94.5	88.6	88.5	96.5	94.2	96.0	9	A-chicken-K1600657-USA-2019
10	5.7	5.5	2.7	6.2	11.9	12.4	35.8	1.3	0.4		89.4	89.2	97.8	99.0	99.1	97.9	85.8	88.5	99.3	70.3	94.7	88.5	88.3	96.6	94.4	96.2	10	A-chicken-05682-USA-2015
11	11.9	11.7	12.2	11.9	5.5	6.0	36.5	11.7	11.5	11.3		97.5	89.2	88.8	88.9	89.4	88.6	96.3	89.2	70.2	89.1	94.1	94.5	89.5	88.9	89.8	11	A-chicken-D4-Canada-2020
12	12.2	12.0	12.0	12.4	6.2	6.7	35.8	11.5	11.3	11.5	2.4		89.4	88.6	88.8	89.5	88.9	96.2	89.2	70.5	88.9	93.5	93.9	89.4	88.9	89.7	12	A-chicken-AVS-B-Hungary-2010
13	5.7	5.5	1.9	6.2	11.7	12.2	36.2	2.9	2.3	2.1	11.5	11.3		97.2	97.3	99.7	86.3	88.6	97.5	70.3	95.3	88.3	88.2	96.8	95.0	96.8	13	A-chicken-LY383-China-2018
14	6.2	6.0	3.3	6.7	12.6	13.1	36.5	1.9	1.0	0.9	12.0	12.2	2.7		98.8	97.3	85.4	87.9	98.7	69.9	94.1	87.9	87.7	96.0	93.9	95.6	14	A-chicken-S5-USA-2024
15	6.0	5.9	3.2	6.5	12.4	12.9	36.2	1.8	0.9	0.7	11.9	12.0	2.6	1.0		97.5	85.5	88.0	98.8	70.0	94.4	88.0	87.9	96.2	94.1	95.7	15	A-chicken-94594-USA-2023
16	5.5	5.4	2.1	6.0	11.5	12.0	36.0	2.7	2.1	1.9	11.3	11.2	0.1	2.6	2.4		86.1	88.5	97.6	70.5	95.4	88.5	88.3	96.9	94.8	96.9	16	A-chicken-SD26-China-2022
17	15.8	15.6	15.2	16.0	13.4	13.6	32.7	15.2	15.8	15.6	12.2	11.9	15.1	16.2	16.0	15.2		88.5	86.1	71.5	85.5	87.1	87.1	85.8	85.8	86.4	17	A-chicken-3211-Hungary-2016
18	12.7	12.6	12.2	12.7	6.0	6.5	36.5	12.9	12.6	12.4	3.6	3.8	12.2	13.1	12.9	12.4	12.4		88.5	70.2	88.8	93.6	94.1	88.8	88.8	89.4	18	A-chicken-D10-Canada-2020
19	6.0	5.9	3.0	6.5	11.9	12.4	35.3	1.3	0.7	0.6	11.5	11.5	2.4	1.2	1.0	2.3	15.2	12.4		70.6	94.4	88.5	88.3	96.3	94.2	95.9	19	A-chicken-D2-Canada-2020
20	34.8	34.6	36.5	35.1	34.8	35.5	5.2	35.8	36.2	36.0	36.5	36.0	36.0	36.7	36.5	35.8	34.1	36.5	35.5		69.7	70.8	70.9	70.2	69.7	70.5	20	A-chicken-924-Hungary-2016
21	7.0	6.8	5.7	7.5	12.0	12.6	36.7	6.0	5.5	5.4	11.7	11.9	4.7	6.0	5.7	4.6	16.0	12.0	5.7	36.9		88.5	88.0	96.6	93.6	97.0	21	FJNP01
22	12.6	12.4	13.1	12.6	1.2	1.6	36.3	12.4	12.1	12.2	5.9	6.5	12.4	12.9	12.8	12.2	13.8	6.3	12.2	35.4	12.2		99.1	88.8	89.1	89.1	22	FJNP02
23	12.7	12.6	13.4	12.7	1.5	1.9	36.2	12.7	12.4	12.6	5.5	6.2	12.7	13.3	13.1	12.6	14.0	6.0	12.6	35.3	12.9	0.6		88.6	88.5	88.9	23	FJNP03
24	5.4	5.1	3.6	5.9	11.0	11.5	36.3	3.8	3.3	3.2	11.0	11.2	3.0	3.8	3.6	2.9	15.4	11.9	3.5	36.1	3.2	11.7	12.1		94.7	97.5	24	FJNP04
25	5.4	5.2	5.9	5.9	11.7	12.2	37.2	5.7	5.9	5.7	11.9	11.9	5.1	6.2	6.0	5.2	15.6	12.0	5.9	36.9	6.5	11.5	12.4	5.2		94.5	25	FJNP05
26	5.4	5.2	4.1	5.9	10.8	11.3	35.5	4.3	4.0	3.8	10.8	11.0	3.2	4.4	4.3	3.0	14.9	11.3	4.1	35.8	2.9	11.5	11.9	2.3	5.5		26	FJNP06
	1	2	3	4	5	6	7	8	9	10	11	12	13	14	15	16	17	18	19	20	21	22	23	24	25	26		

(1-e) μB Nucleotide sequence identity analysis

Percent Identity

Divergence

	1	2	3	4	5	6	7	8	9	10	11	12	13	14	15	16	17	18	19	20	21	22	23	24	25	26		
1		99.8	73.5	99.1	72.6	73.0	81.7	73.2	72.8	73.0	73.6	72.7	81.5	72.3	72.4	81.5	82.1	73.5	73.2	81.3	72.7	72.6	81.5	72.5	72.7	72.7	1	A-chicken-S1133-China-2014
2	0.2		73.5	99.3	72.6	73.0	81.7	73.2	72.9	73.1	73.6	72.7	81.5	72.3	72.4	81.6	82.2	73.5	73.2	81.3	72.7	72.6	81.6	72.5	72.6	72.6	2	A-chicken-1733-China-2014
3	33.5	33.4		73.4	89.7	89.7	73.5	90.9	90.2	89.6	90.9	90.5	73.4	90.2	90.3	73.4	73.9	90.7	90.9	73.1	89.5	89.8	73.5	89.9	89.8	89.7	3	A-chicken-526-China-2014
4	0.9	0.7	33.7		72.4	72.7	81.5	72.9	72.5	73.0	73.4	72.4	81.3	72.1	72.1	81.4	81.9	73.3	73.0	81.0	72.6	72.4	81.5	72.3	72.5	72.5	4	A-chicken-GX2010-China-2015
5	34.9	34.9	11.3	35.4		97.1	72.4	90.9	88.1	87.1	88.3	88.6	72.5	87.8	87.9	72.5	72.8	88.1	88.4	72.1	97.0	98.0	72.5	97.3	97.6	97.0	5	A-chicken-PHC-China-2022
6	34.3	34.4	11.4	34.7	3.0		72.4	90.4	88.1	87.1	88.3	88.1	72.5	87.4	87.5	72.6	72.9	88.1	88.0	72.2	96.5	96.7	72.4	96.8	96.4	96.5	6	A-chicken-202305-China-2024
7	21.8	21.8	33.4	22.1	35.3	35.2		72.9	72.4	73.1	73.0	72.9	94.9	72.5	72.6	95.0	91.1	72.8	73.4	84.9	72.3	72.5	94.9	72.1	72.5	72.2	7	A-chicken-AHZJ19-China-2021
8	34.0	33.9	9.9	34.5	9.9	10.5	34.4		88.5	87.9	88.8	89.0	73.0	88.7	88.8	73.0	73.3	88.4	88.9	72.2	90.5	90.7	72.9	90.8	90.7	90.6	8	A-chicken-01224A-USA-2016
9	34.6	34.4	10.6	35.0	13.3	13.3	35.1	12.8		87.9	93.2	89.5	72.5	88.4	88.5	72.5	72.9	93.0	88.3	72.8	88.3	88.1	72.8	88.5	88.2	88.4	9	A-chicken-K1600657-USA-2019
10	34.3	34.2	11.5	34.2	14.6	14.7	33.9	13.6	13.5		88.2	88.6	73.3	87.7	87.8	73.3	73.6	88.0	87.5	72.8	87.7	86.9	73.3	87.5	87.6	87.3	10	A-chicken-05682-USA-2015
11	33.2	33.2	10.0	33.5	13.1	13.1	34.2	12.5	7.1	13.2		90.2	72.9	89.2	89.3	72.9	73.5	99.5	88.8	73.1	88.4	88.2	73.2	89.0	88.5	88.8	11	A-chicken-D4-Canada-2020
12	34.9	34.9	10.4	35.3	12.7	13.3	34.3	12.3	11.6	12.7	10.8		72.9	95.2	95.3	72.9	73.4	89.9	89.2	72.9	88.8	88.1	72.7	88.6	88.8	88.5	12	A-chicken-AVS-B-Hungary-2010
13	22.1	22.0	33.5	22.3	35.1	35.0	5.3	34.3	34.9	33.6	34.3	34.4		72.5	72.5	99.9	91.2	72.8	73.3	84.7	72.3	72.6	94.6	72.1	72.5	72.2	13	A-chicken-LY383-China-2018
14	35.5	35.5	10.8	35.9	13.7	14.2	35.0	12.6	12.8	13.8	11.9	5.1	35.1		99.9	72.5	72.8	89.0	88.8	72.8	87.9	87.5	72.1	87.9	87.9	87.7	14	A-chicken-S5-USA-2024
15	35.4	35.4	10.6	35.8	13.6	14.1	34.9	12.4	12.7	13.7	11.8	4.9	35.0	0.1		72.5	72.8	89.1	88.9	72.8	88.0	87.6	72.2	88.0	88.0	87.8	15	A-chicken-94594-USA-2023
16	22.1	22.0	33.5	22.3	35.1	35.0	5.3	34.2	34.9	33.6	34.3	34.4	0.1	35.0	35.0		91.2	72.8	73.3	84.7	72.4	72.6	94.6	72.1	72.5	72.2	16	A-chicken-SD26-China-2022
17	21.2	21.0	32.7	21.5	34.6	34.3	9.7	33.6	34.2	33.0	33.3	33.5	9.6	34.5	34.4	9.6		73.4	74.0	85.4	72.9	72.9	90.7	72.6	72.9	72.6	17	A-chicken-3211-Hungary-2016
18	33.4	33.4	10.2	33.7	13.3	13.4	34.5	12.9	7.4	13.5	0.5	11.1	34.6	12.2	12.1	34.5	33.5		88.5	73.0	88.3	88.1	73.1	88.8	88.3	88.6	18	A-chicken-D10-Canada-2020
19	34.0	34.0	9.9	34.4	13.0	13.5	33.5	12.4	13.1	14.1	12.5	11.9	33.7	12.5	12.3	33.7	32.5	12.8		73.1	88.3	88.1	73.5	88.2	88.2	88.5	19	A-chicken-D2-Canada-2020
20	21.7	21.7	33.2	22.1	34.8	34.7	16.7	34.6	33.5	33.7	33.1	33.5	16.9	33.6	33.6	16.9	16.0	33.4	33.3		72.1	72.0	85.0	72.1	72.2	72.0	20	A-chicken-924-Hungary-2016
21	34.8	34.8	11.6	35.0	3.1	3.6	35.4	10.4	13.0	13.8	12.9	12.4	35.4	13.6	13.4	35.3	34.4	13.1	13.0	34.8		96.6	72.3	98.6	97.4	98.8	21	FJNP01
22	35.0	35.0	11.2	35.3	2.1	3.4	35.1	10.2	13.3	14.8	13.2	13.3	34.9	14.1	14.0	34.9	34.4	13.3	13.4	35.1	3.5		72.6	96.8	99.1	96.6	22	FJNP02
23	22.1	22.0	33.4	22.1	35.1	35.3	5.3	34.5	34.4	33.6	33.9	34.6	5.7	35.7	35.6	5.7	10.1	34.1	33.3	16.6	35.4	34.9		72.2	72.6	72.2	23	FJNP03
24	35.2	35.2	11.2	35.5	2.8	3.3	35.8	10.1	12.8	14.1	12.2	12.7	35.8	13.6	13.4	35.8	34.9	12.4	13.2	34.8	1.4	3.3	35.7		97.2	98.9	24	FJNP04
25	34.9	35.0	11.2	35.2	2.4	3.7	35.1	10.2	13.2	13.9	12.9	12.5	35.1	13.5	13.4	35.0	34.4	13.1	13.3	34.7	2.7	0.9	34.9	2.9		96.9	25	FJNP05
26	34.9	35.0	11.4	35.1	3.1	3.6	35.7	10.3	12.8	14.3	12.5	12.8	35.7	13.8	13.7	35.6	34.8	12.6	12.9	35.0	1.2	3.6	35.6	1.1	3.2		26	FJNP06
	1	2	3	4	5	6	7	8	9	10	11	12	13	14	15	16	17	18	19	20	21	22	23	24	25	26		

(1-f) μB amino sequence identity analysis

Percent Identity

Divergence

	1	2	3	4	5	6	7	8	9	10	11	12	13	14	15	16	17	18	19	20	21	22	23	24	25	26		
1		99.1	94.1	98.5	88.6	88.2	70.9	94.4	94.2	94.4	88.9	88.6	94.4	93.9	94.1	94.5	85.7	88.2	94.1	71.0	93.2	88.2	88.2	94.5	94.7	94.7	1	A-chicken-S1133-China-2014
2	0.7		94.2	98.7	88.8	88.3	71.0	94.5	94.4	94.5	89.1	88.8	94.5	94.1	94.2	94.7	85.8	88.3	94.2	71.2	93.4	88.3	88.3	94.8	94.8	94.8	2	A-chicken-1733-China-2014
3	6.0	5.9		93.6	88.2	87.7	70.0	96.5	97.0	97.2	88.6	88.8	97.9	96.6	96.8	97.8	86.1	88.6	96.9	70.0	94.4	87.7	87.6	96.2	94.2	95.9	3	A-chicken-526-China-2014
4	1.3	1.2	6.5		88.6	88.2	70.8	93.9	93.8	93.9	88.9	88.5	93.9	93.5	93.6	94.1	85.5	88.2	93.6	70.9	92.8	88.2	88.2	94.1	94.2	94.2	4	A-chicken-GX2010-China-2015
5	12.2	12.0	12.7	12.2		99.4	70.6	88.8	89.1	88.9	94.5	93.9	89.1	88.3	88.5	89.2	87.6	94.1	88.9	71.2	88.8	98.5	98.4	89.5	89.1	89.8	5	A-chicken-PHC-China-2022
6	12.7	12.6	13.3	12.7	0.4		70.5	88.3	88.6	88.5	94.1	93.5	88.6	87.9	88.0	88.8	87.4	93.6	88.5	70.8	88.3	98.1	97.9	89.1	88.6	89.4	6	A-chicken-202305-China-2024
7	35.1	34.8	36.5	35.3	35.8	36.0		70.6	70.3	70.5	70.2	70.6	70.2	70.0	70.2	70.3	72.4	70.2	70.8	91.8	69.9	70.2	70.3	70.0	69.6	70.6	7	A-chicken-AHZJ19-China-2021
8	5.7	5.5	3.5	6.2	12.0	12.6	35.5		98.4	98.5	89.1	89.2	97.0	97.9	98.1	97.2	86.1	88.0	98.5	70.5	94.1	88.3	88.2	96.0	94.4	95.7	8	A-chicken-01224A-USA-2016
9	5.9	5.7	2.9	6.3	11.7	12.2	36.0	1.5		99.4	89.2	89.4	97.6	98.8	99.0	97.8	85.7	88.3	99.1	70.2	94.5	88.6	88.5	96.5	94.2	96.0	9	A-chicken-K1600657-USA-2019
10	5.7	5.5	2.7	6.2	11.9	12.4	35.8	1.3	0.4		89.4	89.2	97.8	99.0	99.1	97.9	85.8	88.5	99.3	70.3	94.7	88.5	88.3	96.6	94.4	96.2	10	A chicken 05682 USA 2015
11	11.9	11.7	12.2	11.9	5.5	6.0	36.5	11.7	11.5	11.3		97.5	89.2	88.8	88.9	89.4	88.6	96.3	89.2	70.2	89.1	94.1	94.5	89.5	88.9	89.8	11	A chicken D4 Canada 2020
12	12.2	12.0	12.0	12.4	6.2	6.7	35.8	11.5	11.3	11.5	2.4		89.4	88.6	88.8	89.5	88.9	96.2	89.2	70.5	88.9	93.5	93.9	89.4	88.9	89.7	12	A-chicken-AVS-B-Hungary-2010
13	5.7	5.5	1.9	6.2	11.7	12.2	36.2	2.9	2.3	2.1	11.5	11.3		97.2	97.3	99.7	86.3	88.6	97.5	70.3	95.3	88.3	88.2	96.8	95.0	96.8	13	A-chicken-LY383-China-2018
14	6.2	6.0	3.3	6.7	12.6	13.1	36.5	1.9	1.0	0.9	12.0	12.2	2.7		98.8	97.3	85.4	87.9	98.7	69.9	94.1	87.9	87.7	96.0	93.9	95.6	14	A-chicken-S5-USA-2024
15	6.0	5.9	3.2	6.5	12.4	12.9	36.2	1.8	0.9	0.7	11.9	12.0	2.6	1.0		97.5	85.5	88.0	98.8	70.0	94.4	88.0	87.9	96.2	94.1	95.7	15	A-chicken-94594-USA-2023
16	5.5	5.4	2.1	6.0	11.5	12.0	36.0	2.7	2.1	1.9	11.3	11.2	0.1	2.6	2.4		86.1	88.5	97.6	70.5	95.4	88.5	88.3	96.9	94.8	96.9	16	A-chicken-SD26-China-2022
17	15.8	15.6	15.2	16.0	13.4	13.6	32.7	15.2	15.8	15.6	12.2	11.9	15.1	16.2	16.0	15.2		88.5	86.1	71.5	85.5	87.1	87.1	85.8	85.8	86.4	17	A-chicken-3211-Hungary-2016
18	12.7	12.6	12.2	12.7	6.0	6.5	36.5	12.9	12.6	12.4	3.6	3.8	12.2	13.1	12.9	12.4	12.4		88.5	70.2	88.8	93.6	94.1	88.8	88.8	89.4	18	A-chicken-D10-Canada-2020
19	6.0	5.9	3.0	6.5	11.9	12.4	35.3	1.3	0.7	0.6	11.5	11.5	2.4	1.2	1.0	2.3	15.2	12.4		70.6	94.4	88.5	88.3	96.3	94.2	95.9	19	A-chicken-D2-Canada-2020
20	34.8	34.6	36.5	35.1	34.8	35.5	5.2	35.8	36.2	36.0	36.5	36.0	36.0	36.7	36.5	35.8	34.1	36.5	35.5		69.7	70.8	70.9	70.2	69.7	70.5	20	A-chicken-924-Hungary-2016
21	7.0	6.8	5.7	7.5	12.0	12.6	36.7	6.0	5.5	5.4	11.7	11.9	4.7	6.0	5.7	4.6	16.0	12.0	5.7	36.9		88.5	88.0	96.6	93.6	97.0	21	FJNP01
22	12.6	12.4	13.1	12.6	1.2	1.6	36.3	12.4	12.1	12.2	5.9	6.5	12.4	12.9	12.8	12.2	13.8	6.3	12.2	35.4	12.2		99.1	88.8	89.1	89.1	22	FJNP02
23	12.7	12.6	13.4	12.7	1.5	1.9	36.2	12.7	12.4	12.6	5.5	6.2	12.7	13.3	13.1	12.6	14.0	6.0	12.6	35.3	12.9	0.6		88.6	88.5	88.9	23	FJNP03
24	5.4	5.1	3.6	5.9	11.0	11.5	36.3	3.8	3.3	3.2	11.0	11.2	3.0	3.8	3.6	2.9	15.4	11.9	3.5	36.1	3.2	11.7	12.1		94.7	97.5	24	FJNP04
25	5.4	5.2	5.9	5.9	11.7	12.2	37.2	5.7	5.9	5.7	11.9	11.9	5.1	6.2	6.0	5.2	15.6	12.0	5.9	36.9	6.5	11.5	12.4	5.2		94.5	25	FJNP05
26	5.4	5.2	4.1	5.9	10.8	11.3	35.5	4.3	4.0	3.8	10.8	11.0	3.2	4.4	4.3	3.0	14.9	11.3	4.1	35.8	2.9	11.5	11.9	2.3	5.5		26	FJNP06
	1	2	3	4	5	6	7	8	9	10	11	12	13	14	15	16	17	18	19	20	21	22	23	24	25	26		

(1-g) λC Nucleotide sequence identity analysis

Percent Identity

Divergence

	1	2	3	4	5	6	7	8	9	10	11	12	13	14	15	16	17	18	19	20	21	22	23	24	25	26		
1		99.8	73.5	99.1	72.6	73.0	81.7	73.2	72.8	73.0	73.6	72.7	81.5	72.3	72.4	81.5	82.1	73.5	73.2	81.3	72.7	72.6	81.5	72.5	72.7	72.7	1	A-chicken-S1133-China-2014
2	0.2		73.5	99.3	72.6	73.0	81.7	73.2	72.9	73.1	73.6	72.7	81.5	72.3	72.4	81.6	82.2	73.5	73.2	81.3	72.7	72.6	81.6	72.5	72.6	72.6	2	A-chicken-1733-China-2014
3	33.5	33.4		73.4	89.7	89.7	73.5	90.9	90.2	89.6	90.9	90.5	73.4	90.2	90.3	73.4	73.9	90.7	90.9	73.1	89.5	89.8	73.5	89.9	89.8	89.7	3	A-chicken-526-China-2014
4	0.9	0.7	33.7		72.4	72.7	81.5	72.9	72.5	73.0	73.4	72.4	81.3	72.1	72.1	81.4	81.9	73.3	73.0	81.0	72.6	72.4	81.5	72.3	72.5	72.5	4	A-chicken-GX2010-China-2015
5	34.9	34.9	11.3	35.4		97.1	72.4	90.9	88.1	87.1	88.3	88.6	72.5	87.8	87.9	72.5	72.8	88.1	88.4	72.1	97.0	98.0	72.5	97.3	97.6	97.0	5	A-chicken-PHC-China-2022
6	34.3	34.4	11.4	34.7	3.0		72.4	90.4	88.1	87.1	88.3	88.1	72.5	87.4	87.5	72.6	72.9	88.1	88.0	72.2	96.5	96.7	72.4	96.8	96.4	96.5	6	A-chicken-202305-China-2024
7	21.8	21.8	33.4	22.1	35.3	35.2		72.9	72.4	73.1	73.0	72.9	94.9	72.5	72.6	95.0	91.1	72.8	73.4	84.9	72.3	72.5	94.9	72.1	72.5	72.2	7	A-chicken-AHZJ19-China-2021
8	34.0	33.9	9.9	34.5	9.9	10.5	34.4		88.5	87.9	88.8	89.0	73.0	88.7	88.8	73.0	73.3	88.4	88.9	72.2	90.5	90.7	72.9	90.8	90.7	90.6	8	A-chicken-01224A-USA-2016
9	34.6	34.4	10.6	35.0	13.3	13.3	35.1	12.8		87.9	93.2	89.5	72.5	88.4	88.5	72.5	72.9	93.0	88.3	72.8	88.3	88.1	72.8	88.5	88.2	88.4	9	A-chicken-K1600657-USA-2019
10	34.3	34.2	11.5	34.2	14.6	14.7	33.9	13.6	13.5		88.2	88.6	73.3	87.7	87.8	73.3	73.6	88.0	87.5	72.8	87.7	86.9	73.3	87.5	87.6	87.3	10	A chicken 05682 USA 2015
11	33.2	33.2	10.0	33.5	13.1	13.1	34.2	12.5	7.1	13.2		90.2	72.9	89.2	89.3	72.9	73.5	99.5	88.8	73.1	88.4	88.2	73.2	89.0	88.5	88.8	11	A chicken D4 Canada 2020
12	34.9	34.9	10.4	35.3	12.7	13.3	34.3	12.3	11.6	12.7	10.8		72.9	95.2	95.3	72.9	73.4	89.9	89.2	72.9	88.8	88.1	72.7	88.6	88.8	88.5	12	A-chicken-AVS-B-Hungary-2010
13	22.1	22.0	33.5	22.3	35.1	35.0	5.3	34.3	34.9	33.6	34.3	34.4		72.5	72.5	99.9	91.2	72.8	73.3	84.7	72.3	72.6	94.6	72.1	72.5	72.2	13	A-chicken-LY383-China-2018
14	35.5	35.5	10.8	35.9	13.7	14.2	35.0	12.6	12.8	13.8	11.9	5.1	35.1		99.9	72.5	72.8	89.0	88.8	72.8	87.9	87.5	72.1	87.9	87.9	87.7	14	A-chicken-S5-USA-2024
15	35.4	35.4	10.6	35.8	13.6	14.1	34.9	12.4	12.7	13.7	11.8	4.9	35.0	0.1		72.5	72.8	89.1	88.9	72.8	88.0	87.6	72.2	88.0	88.0	87.8	15	A-chicken-94594-USA-2023
16	22.1	22.0	33.5	22.3	35.1	35.0	5.3	34.2	34.9	33.6	34.3	34.4	0.1	35.0	35.0		91.2	72.8	73.3	84.7	72.4	72.6	94.6	72.1	72.5	72.2	16	A-chicken-SD26-China-2022
17	21.2	21.0	32.7	21.5	34.6	34.3	9.7	33.6	34.2	33.0	33.3	33.5	9.6	34.5	34.4	9.6		73.4	74.0	85.4	72.9	72.9	90.7	72.6	72.9	72.6	17	A-chicken-3211-Hungary-2016
18	33.4	33.4	10.2	33.7	13.3	13.4	34.5	12.9	7.4	13.5	0.5	11.1	34.6	12.2	12.1	34.5	33.5		88.5	73.0	88.3	88.1	73.1	88.8	88.3	88.6	18	A-chicken-D10-Canada-2020
19	34.0	34.0	9.9	34.4	13.0	13.5	33.5	12.4	13.1	14.1	12.5	11.9	33.7	12.5	12.3	33.7	32.5	12.8		73.1	88.3	88.1	73.5	88.2	88.2	88.5	19	A-chicken-D2-Canada-2020
20	21.7	21.7	33.2	22.1	34.8	34.7	16.7	34.6	33.5	33.7	33.1	33.5	16.9	33.6	33.6	16.9	16.0	33.4	33.3		72.1	72.0	85.0	72.1	72.2	72.0	20	A-chicken-924-Hungary-2016
21	34.8	34.8	11.6	35.0	3.1	3.6	35.4	10.4	13.0	13.8	12.9	12.4	35.4	13.6	13.4	35.3	34.4	13.1	13.0	34.8		96.6	72.3	98.6	97.4	98.8	21	FJNP01
22	35.0	35.0	11.2	35.3	2.1	3.4	35.1	10.2	13.3	14.8	13.2	13.3	34.9	14.1	14.0	34.9	34.4	13.3	13.4	35.1	3.5		72.6	96.8	99.1	96.6	22	FJNP02
23	22.1	22.0	33.4	22.1	35.1	35.3	5.3	34.5	34.4	33.6	33.9	34.6	5.7	35.7	35.6	5.7	10.1	34.1	33.3	16.6	35.4	34.9		72.2	72.6	72.2	23	FJNP03
24	35.2	35.2	11.2	35.5	2.8	3.3	35.8	10.1	12.8	14.1	12.2	12.7	35.8	13.6	13.4	35.8	34.9	12.4	13.2	34.8	1.4	3.3	35.7		97.2	98.9	24	FJNP04
25	34.9	35.0	11.2	35.2	2.4	3.7	35.1	10.2	13.2	13.9	12.9	12.5	35.1	13.5	13.4	35.0	34.4	13.1	13.3	34.7	2.7	0.9	34.9	2.9		96.9	25	FJNP05
26	34.9	35.0	11.4	35.1	3.1	3.6	35.7	10.3	12.8	14.3	12.5	12.8	35.7	13.8	13.7	35.6	34.8	12.6	12.9	35.0	1.2	3.6	35.6	1.1	3.2		26	FJNP06
	1	2	3	4	5	6	7	8	9	10	11	12	13	14	15	16	17	18	19	20	21	22	23	24	25	26		

(1-h) λC amino sequence identity analysis

Percent Identity

Divergence

	1	2	3	4	5	6	7	8	9	10	11	12	13	14	15	16	17	18	19	20	21	22	23	24	25	26		
1		99.7	83.7	98.9	83.7	83.6	92.9	83.7	84.0	83.7	84.1	84.1	92.7	83.9	84.0	92.8	93.5	84.1	84.4	92.8	84.1	83.5	92.9	83.8	83.7	83.9	1	A-chicken-S1133-China-2014
2	0.2		83.7	99.0	83.7	83.7	93.0	83.7	84.1	83.8	84.2	84.1	92.8	84.0	84.1	92.8	93.5	84.1	84.4	92.8	84.2	83.6	93.0	83.9	83.8	84.0	2	A-chicken-1733-China-2014
3	18.4	18.3		83.4	96.6	96.4	83.3	95.7	96.7	95.7	96.3	96.2	83.4	96.3	96.5	83.4	83.3	96.3	96.8	83.3	95.6	96.2	83.1	96.1	96.3	95.9	3	A-chicken-526-China-2014
4	1.0	0.9	18.7		83.5	83.4	92.4	83.4	83.7	83.6	83.9	83.8	92.1	83.7	83.7	92.2	92.9	83.8	84.1	92.3	84.0	83.4	92.4	83.7	83.6	83.7	4	A-chicken-GX2010-China-2015
5	18.4	18.3	3.4	18.6		99.0	83.9	97.2	96.2	95.3	95.8	96.1	83.9	95.8	96.0	83.9	83.7	95.8	96.4	83.6	98.4	99.1	83.8	98.5	99.1	98.7	5	A-chicken-PHC-China-2022
6	18.5	18.4	3.6	18.7	0.9		83.8	97.0	95.9	94.9	95.5	95.9	83.8	95.5	95.6	83.8	83.7	95.5	96.4	83.5	98.1	98.6	83.7	98.2	98.8	98.4	6	A-chicken-202305-China-2024
7	7.4	7.3	18.9	8.0	18.1	18.2		83.4	83.8	83.4	83.8	83.9	98.4	83.8	83.9	98.4	97.4	83.8	84.3	93.9	83.9	83.7	98.9	83.5	83.9	83.7	7	A-chicken-AHZJ19-China-2021
8	18.4	18.4	4.3	18.8	2.8	3.0	18.7		95.5	94.6	95.2	95.0	83.4	94.8	94.9	83.4	83.3	95.0	95.6	83.4	96.2	96.9	83.3	96.3	96.9	96.5	8	A-chicken-01224A-USA-2016
9	17.8	17.7	3.2	18.1	3.7	4.0	18.0	4.4		96.1	96.8	96.0	83.9	96.0	96.2	83.9	83.7	96.7	96.7	83.8	95.8	95.9	83.7	95.7	96.2	95.9	9	A-chicken-K1600657-USA-2019
10	18.2	18.1	4.3	18.4	4.7	5.1	18.7	5.4	3.7		95.9	95.4	83.5	95.6	95.8	83.5	83.5	95.7	95.9	83.4	94.9	94.9	83.3	94.9	95.3	94.9	10	A-chicken-05682-USA-2015
11	17.8	17.7	3.7	18.1	4.2	4.6	18.2	4.9	3.0	4.1		95.9	83.7	95.8	96.0	83.7	83.8	99.4	96.4	83.7	95.2	95.4	83.6	95.6	95.6	95.5	11	A-chicken-D4-Canada-2020
12	17.9	17.8	3.8	18.2	3.9	4.2	18.1	5.1	3.8	4.6	4.2		84.1	97.7	97.9	84.1	84.0	95.7	96.3	83.6	95.5	95.9	83.7	95.3	96.1	95.5	12	A-chicken-AVS-B-Hungary-2010
13	7.6	7.5	18.7	8.2	18.1	18.2	1.6	18.7	17.9	18.5	18.3	17.8		84.0	84.1	99.7	97.3	83.7	84.3	94.2	84.0	83.9	98.4	83.5	84.1	83.7	13	A-chicken-LY383-China-2018
14	18.1	18.0	3.7	18.4	4.2	4.6	18.2	5.3	3.8	4.3	4.2	2.2	18.0		99.8	84.0	83.9	95.8	96.0	83.4	95.0	95.6	83.7	95.2	95.6	95.2	14	A-chicken-S5-USA-2024
15	18.0	17.9	3.5	18.3	4.1	4.5	18.1	5.2	3.7	4.2	4.1	2.1	17.9	0.2		84.1	84.0	96.0	96.2	83.5	95.2	95.7	83.7	95.3	95.8	95.3	15	A-chicken-94594-USA-2023
16	7.5	7.5	18.7	8.1	18.1	18.2	1.5	18.7	17.9	18.5	18.3	17.8	0.2	18.0	17.9		97.4	83.7	84.3	94.3	84.0	83.9	98.4	83.5	84.1	83.7	16	A-chicken-SD26-China-2022
17	6.8	6.7	18.9	7.4	18.3	18.4	2.5	18.9	18.1	18.5	18.2	18.0	2.7	18.1	18.0	2.6		83.8	84.4	94.2	84.1	83.6	97.1	83.7	83.8	83.8	17	A-chicken-3211-Hungary-2016
18	17.9	17.8	3.8	18.2	4.2	4.6	18.2	5.1	3.1	4.3	0.5	4.3	18.3	4.2	4.1	18.3	18.2		96.3	83.7	95.1	95.6	83.6	95.6	95.6	95.4	18	A-chicken-D10-Canada-2020
19	17.5	17.4	3.2	17.8	3.6	3.6	17.6	4.4	3.1	4.1	3.6	3.7	17.6	4.0	3.8	17.6	17.4	3.7		84.2	96.1	96.3	84.1	96.0	96.3	96.4	19	A-chicken-D2-Canada-2020
20	6.9	6.8	18.2	7.4	17.8	17.9	5.6	18.0	17.4	17.9	17.7	17.8	5.3	18.0	17.9	5.2	5.3	17.7	17.0		83.3	83.6	94.2	83.2	83.8	83.2	20	A-chicken-924-Hungary-2016
21	17.8	17.7	4.5	18.0	1.5	1.9	18.1	3.8	4.1	5.1	4.9	4.6	18.0	5.1	4.9	18.0	17.9	5.0	3.9	18.2		98.1	83.7	98.7	98.6	99.1	21	FJNP01
22	18.6	18.5	3.8	18.8	0.9	1.3	18.3	3.1	4.0	5.1	4.7	4.2	18.1	4.5	4.3	18.1	18.5	4.5	3.8	17.8	1.8		83.7	98.2	99.2	98.4	22	FJNP02
23	7.4	7.3	19.1	8.0	18.2	18.4	1.0	18.9	18.1	18.8	18.5	18.3	1.6	18.4	18.3	1.5	2.9	18.5	17.8	5.4	18.3	18.3		83.4	83.9	83.5	23	FJNP03
24	18.2	18.1	3.9	18.4	1.4	1.7	18.6	3.7	4.2	5.1	4.4	4.7	18.6	4.9	4.7	18.6	18.4	4.5	4.1	18.3	1.3	1.7	18.8		98.4	99.0	24	FJNP04
25	18.3	18.2	3.8	18.5	0.8	1.2	18.1	3.1	3.7	4.7	4.5	3.9	17.8	4.4	4.2	17.8	18.2	4.5	3.8	17.5	1.3	0.7	18.1	1.5		98.6	25	FJNP05
26	18.1	18.0	4.2	18.3	1.3	1.6	18.4	3.5	4.0	5.1	4.6	4.6	18.4	4.9	4.7	18.4	18.2	4.7	3.6	18.3	0.8	1.6	18.6	0.9	1.3		26	FJNP06
	1	2	3	4	5	6	7	8	9	10	11	12	13	14	15	16	17	18	19	20	21	22	23	24	25	26		

(1-i) P10 Nucleotide sequence identity analysis

Percent Identity

Divergence

	1	2	3	4	5	6	7	8	9	10	11	12	13	14	15	16	17	18	19	20	21	22	23		
1		99.3	87.9	99.0	68.7	68.0	62.3	67.0	63.0	64.3	69.4	69.4	69.4	69.4	64.3	65.0	63.3	84.2	67.7	63.3	62.3	70.4	63.6	1	A-chicken-S1133-China-2014
2	0.7		87.5	99.0	68.0	67.3	61.3	66.0	62.0	63.6	69.7	69.7	69.7	69.7	65.3	65.3	62.3	83.5	67.0	63.6	61.6	70.4	64.3	2	A-chicken-1733-China-2014
3	11.8	12.2		86.9	68.0	67.0	63.0	63.3	63.3	61.3	70.0	70.7	70.7	70.0	65.7	65.7	61.6	83.2	66.7	63.6	59.3	70.4	65.3	3	A-chicken-526-China-2014
4	1.0	1.0	12.6		69.4	68.7	61.6	66.3	61.3	63.6	69.0	69.0	69.0	69.0	64.6	64.6	62.6	83.5	68.4	63.6	61.6	69.7	64.0	4	A-chicken-GX2010-China-2015
5	32.4	32.9	34.6	31.8		99.0	69.0	72.0	70.0	71.0	71.3	71.7	71.7	71.3	67.0	66.0	65.0	67.0	98.7	72.0	68.0	71.7	68.3	5	A-chicken-PHC-China-2022
6	33.0	33.6	35.7	32.4	1.0		68.7	71.7	70.0	70.3	70.7	71.0	71.0	70.7	66.0	66.3	64.7	66.7	97.7	71.0	67.3	71.0	68.0	6	A-chicken-202305-China-2024
7	39.0	40.3	37.6	39.6	35.0	34.2		90.7	72.0	72.3	72.3	71.3	71.3	72.3	70.7	67.0	71.7	61.6	67.7	73.0	69.3	68.3	70.0	7	A-chicken-AHZJ19-China-2021
8	34.1	35.2	37.6	34.7	30.3	29.5	9.3		73.3	73.7	70.7	70.7	70.7	70.7	69.3	72.7	73.3	63.6	71.0	77.7	70.7	68.0	69.0	8	A-chicken-01224A-USA-2016
9	38.1	39.4	40.9	40.0	31.0	30.7	30.5	27.9		75.3	67.7	68.7	68.7	67.7	76.0	71.3	77.3	64.0	69.0	72.3	71.7	67.7	73.7	9	A-chicken-K1600657-USA-2019
10	35.5	36.7	41.1	36.1	31.7	32.0	29.7	28.9	25.8		72.0	71.7	71.7	72.0	73.7	72.3	76.3	61.6	70.0	73.0	97.3	71.3	73.3	10	A-chicken-D4-Canada-2020
11	31.4	31.4	30.5	32.0	30.5	30.8	30.5	31.4	34.7	29.1		97.7	97.7	100.0	77.7	77.3	73.3	67.3	71.3	69.3	70.0	97.3	75.0	11	A-chicken-LY383-China-2018
12	31.4	31.4	29.9	32.0	29.9	30.2	31.5	31.3	33.5	29.6	2.4		100.0	97.7	77.3	76.3	73.0	68.0	71.7	70.0	69.7	96.7	75.7	12	A-chicken-S5-USA-2024
13	31.4	31.4	29.9	32.0	29.9	30.2	31.5	31.3	33.5	29.6	2.4	0.0		97.7	77.3	76.3	73.0	68.0	71.7	70.0	69.7	96.7	75.7	13	A-chicken-94594-USA-2023
14	31.4	31.4	30.5	32.0	30.5	30.8	30.5	31.4	34.7	29.1	0.0	2.4	2.4		77.7	77.3	73.3	67.3	71.3	69.3	70.0	97.3	75.0	14	A-chicken-SD26-China-2022
15	36.6	35.4	37.9	36.0	35.1	35.4	32.2	33.8	25.0	26.3	23.0	23.5	23.5	23.0		79.7	81.7	62.6	66.3	72.0	70.7	78.0	89.3	15	A-chicken-3211-Hungary-2016
16	34.4	34.4	35.1	35.0	38.0	37.1	38.2	30.2	30.9	30.4	24.1	25.5	25.5	24.1	20.9		81.7	64.3	65.7	70.0	70.0	75.0	76.7	16	A-chicken-D10-Canada-2020
17	37.9	39.2	41.7	38.5	38.2	37.3	31.1	30.0	25.2	24.7	27.8	28.9	28.9	27.8	19.3	20.5		63.3	64.7	71.0	73.3	71.7	80.7	17	A-chicken-924-Hungary-2016
18	16.7	17.1	17.2	17.1	34.8	35.9	40.6	39.9	39.4	40.9	33.9	33.3	33.3	33.9	39.8	37.4	38.6		65.3	65.7	59.3	65.0	65.3	18	FJNP01
19	34.1	34.7	36.4	33.6	1.4	2.4	36.7	32.0	32.7	32.5	31.2	30.6	30.6	31.2	36.9	39.4	39.1	37.2		71.0	67.0	71.7	68.0	19	FJNP02
20	37.6	37.6	38.9	37.0	29.6	29.9	27.9	22.4	28.8	28.7	31.8	31.3	31.3	31.8	29.8	32.8	34.2	36.3	30.7		69.7	70.7	72.7	20	FJNP03
21	38.6	39.8	44.4	39.2	34.6	34.9	33.2	32.4	28.9	2.7	32.0	32.5	32.5	32.0	30.1	33.9	27.8	44.2	35.4	32.1		69.3	70.3	21	FJNP04
22	30.3	30.3	30.4	30.8	29.8	30.1	34.8	34.0	34.7	30.1	2.7	3.4	3.4	2.7	23.4	26.0	29.3	36.8	30.6	31.8	33.0		76.0	22	FJNP05
23	37.6	36.4	37.1	37.0	34.6	34.4	33.3	34.2	27.6	27.4	26.0	25.5	25.5	26.0	11.2	25.2	20.2	35.9	35.9	30.1	30.7	26.0		23	FJNP06
	1	2	3	4	5	6	7	8	9	10	11	12	13	14	15	16	17	18	19	20	21	22	23		

(1-j) P10 amino sequence identity analysis

Percent Identity

Divergence

	1	2	3	4	5	6	7	8	9	10	11	12	13	14	15	16	17	18	19	20	21	22	23		
1		99.0	94.9	98.0	67.7	65.7	67.7	67.7	68.7	67.7	72.7	72.7	72.7	72.7	70.7	69.7	69.7	91.9	66.7	66.7	60.6	73.7	67.7	1	A-chicken-S1133-China-2014
2	0.0		94.9	98.0	67.7	65.7	67.7	67.7	68.7	67.7	72.7	72.7	72.7	72.7	70.7	69.7	69.7	91.9	66.7	66.7	60.6	73.7	67.7	2	A-chicken-1733-China-2014
3	4.2	4.2		93.9	69.7	67.7	68.7	68.7	70.7	68.7	74.7	75.8	75.8	74.7	72.7	71.7	71.7	93.9	68.7	67.7	61.6	74.7	69.7	3	A-chicken-526-China-2014
4	1.0	1.0	5.3		68.7	66.7	66.7	66.7	67.7	66.7	71.7	71.7	71.7	71.7	69.7	68.7	68.7	90.9	67.7	65.7	59.6	72.7	66.7	4	A-chicken-GX2010-China-2015
5	37.6	37.6	32.8	36.0		97.0	76.0	77.0	79.0	79.0	81.0	80.0	80.0	81.0	76.0	74.0	74.0	68.7	97.0	78.0	72.0	80.0	74.0	5	A-chicken-PHC-China-2022
6	41.0	41.0	36.0	39.3	2.0		75.0	76.0	79.0	77.0	79.0	78.0	78.0	79.0	74.0	74.0	74.0	68.7	95.0	76.0	70.0	78.0	73.0	6	A-chicken-202305-China-2024
7	38.1	38.1	34.8	39.8	28.2	29.3		96.0	78.0	78.0	83.0	83.0	83.0	83.0	81.0	76.0	80.0	67.7	75.0	81.0	73.0	83.0	79.0	7	A-chicken-AHZJ19-China-2021
8	38.1	38.1	34.8	39.8	26.7	27.9	3.1		78.0	80.0	82.0	81.0	81.0	82.0	81.0	79.0	82.0	67.7	76.0	83.0	75.0	81.0	79.0	8	A-chicken-01224A-USA-2016
9	38.1	38.1	34.8	39.8	23.9	23.6	25.0	25.0		85.0	83.0	84.0	84.0	83.0	83.0	78.0	80.0	69.7	78.0	81.0	78.0	83.0	83.0	9	A-chicken-K1600657-USA-2019
10	38.1	38.1	34.8	39.8	23.9	26.4	25.0	22.2	15.7		80.0	80.0	80.0	80.0	82.0	85.0	84.0	67.7	78.0	84.0	91.0	81.0	81.0	10	A-chicken-D4-Canada-2020
11	30.1	30.1	25.6	31.6	21.1	23.6	18.3	19.6	18.3	22.2		98.0	98.0	99.0	86.0	84.0	83.0	72.7	81.0	77.0	74.0	98.0	83.0	11	A-chicken-LY383-China-2018
12	30.1	30.1	25.6	31.6	22.5	25.0	18.3	20.9	17.0	22.2	1.0		99.0	98.0	86.0	83.0	84.0	73.7	80.0	78.0	74.0	98.0	84.0	12	A-chicken-S5-USA-2024
13	30.1	30.1	25.6	31.6	22.5	25.0	18.3	20.9	17.0	22.2	1.0	0.0		98.0	86.0	83.0	84.0	73.7	80.0	78.0	74.0	98.0	84.0	13	A-chicken-94594-USA-2023
14	30.1	30.1	25.6	31.6	21.1	23.6	18.3	19.6	18.3	22.2	0.0	1.0	1.0		86.0	84.0	83.0	72.7	81.0	77.0	74.0	98.0	83.0	14	A-chicken-SD26-China-2022
15	33.2	33.2	30.1	34.8	28.2	30.8	20.9	20.9	18.3	19.6	14.5	14.5	14.5	14.5		87.0	92.0	71.7	76.0	80.0	75.0	87.0	93.0	15	A-chicken-3211-Hungary-2016
16	34.8	34.8	30.1	36.4	31.2	30.8	27.9	23.6	25.0	15.7	17.0	18.3	18.3	17.0	13.3		91.0	72.7	74.0	76.0	78.0	83.0	84.0	16	A-chicken-D10-Canada-2020
17	34.8	34.8	31.6	36.4	31.2	30.8	22.2	19.6	22.2	17.0	18.3	17.0	17.0	18.3	7.4	8.6		71.7	74.0	81.0	77.0	83.0	92.0	17	A-chicken-924-Hungary-2016
18	7.5	7.5	5.3	8.7	34.3	34.3	36.4	36.4	36.4	36.4	28.5	28.5	28.5	28.5	31.6	28.5	30.1		67.7	69.7	60.6	72.7	68.7	18	FJNP01
19	39.8	39.8	34.8	38.1	2.1	4.2	29.3	27.9	25.0	25.0	20.9	22.2	22.2	20.9	27.9	30.8	30.8	36.4		77.0	71.0	80.0	74.0	19	FJNP02
20	38.1	38.1	38.1	39.8	25.3	27.9	20.9	18.3	20.9	17.0	26.4	25.0	25.0	26.4	22.2	27.9	20.9	36.4	26.4		78.0	77.0	80.0	20	FJNP03
21	50.9	50.9	47.0	52.9	34.3	37.1	32.4	29.3	25.0	8.6	30.8	30.8	30.8	30.8	29.3	25.0	26.4	48.9	35.5	25.0		75.0	74.0	21	FJNP04
22	30.1	30.1	25.6	31.6	22.5	25.0	18.3	20.9	18.3	20.9	1.0	1.0	1.0	1.0	13.3	18.3	18.3	28.5	22.2	26.4	29.3		83.0	22	FJNP05
23	36.4	36.4	33.2	38.1	31.2	32.4	23.6	23.6	18.3	20.9	18.3	17.0	17.0	18.3	6.3	17.0	7.4	34.8	30.8	22.2	30.8	18.3		23	FJNP06
	1	2	3	4	5	6	7	8	9	10	11	12	13	14	15	16	17	18	19	20	21	22	23		
